# Supplementary material for: Digital multiple health behaviour change intervention targeting online help seekers: protocol for the COACH randomised factorial trial
Source: BMJ Open. 2022 Jul 26;12(7):e061024. doi: 10.1136/bmjopen-2022-061024 (PMC9330315; doi:10.1136/bmjopen-2022-061024)
Supplement: Author's manuscript [file bmjopen-2022-061024.draft_revisions.pdf]

BMJ Open

BMJ Open is committed to open peer review. As part of this commitment we make the peer review history of every article we publish publicly available.

When an article is published we post the peer reviewers' comments and the authors' responses online. We also post the versions of the paper that were used during peer review. These are the versions that the peer review comments apply to.

The versions of the paper that follow are the versions that were submitted during the peer review process. They are not the versions of record or the final published versions. They should not be cited or distributed as the published version of this manuscript.

BMJ Open is an open access journal and the full, final, typeset and author-corrected version of record of the manuscript is available on our site with no access controls, subscription charges or pay-per-view fees (http://bmjopen.bmj.com).

If you have any questions on BMJ Open's open peer review process please email info.bmjopen@bmj.com

BMJ Open

Digital multiple lifestyle behaviour intervention targeting online help seekers: protocol for the Coach randomised factorial trial

Journal:	*BMJ Open*
Manuscript ID	bmjopen-2022-061024
Article Type:	Protocol
Date Submitted by the Author:	17-Jan-2022

Complete List of Authors:	Åsberg, Katarina; Linköping University, Department of Health, Medicine and Caring Sciences<br>Blomqvist, Jenny; Linköping University, Department of Health, Medicine and Caring Sciences<br>Lundgren, Oskar; Linköping University, Department of Health, Medicine and Caring Sciences<br>Henriksson, Hanna; Linköping University, Department of Health, Medicine and Caring Sciences<br>Henriksson, Pontus; Linköping University, Department of Health, Medicine and Caring Sciences<br>Bendtsen, Preben; Linköping University, Department of Health, Medicine and Caring Sciences; Motala Hospital, Department of Medical Specialist<br>Löf, Marie; Linköping University, Department of Health, Medicine and Caring Sciences; Karolinska Institutet, Department of Biosciences and Nutrition<br>Bendtsen, Marcus; Linköping University, Department of Health, Medicine and Caring Sciences

Keywords:	PUBLIC HEALTH, PREVENTIVE MEDICINE, EPIDEMIOLOGY

SCHOLARONE™
Manuscripts

DIGITAL MULTIPLE LIFESTYLE BEHAVIOUR INTERVENTION TARGETING ONLINE HELP SEEKERS: PROTOCOL FOR THE *COACH* RANDOMISED FACTORIAL TRIAL

Katarina Åsberg[1], Jenny Blomqvist[1], Oskar Lundgren[1], Hanna Henriksson[1], Pontus Henriksson[1], Preben Bendtsen[1,2], Marie Löf[1,3], Marcus Bendtsen[1,*]

[1] Department of Health, Medicine and Caring Sciences, Linköping University, Sweden.
[2] Department of Medical Specialist, Motala, Sweden.
[3] Department of Biosciences and Nutrition, Karolinska Institutet, Sweden.

*Corresponding author
Marcus Bendtsen (marcus.bendtsen@liu.se)
Department of Health, Medicine and Caring Sciences, Division of Society and Health
Linköping University, 581 83 Linköping, registrator@liu.se, +46 13 28 10 00

Trial registration: Prospectively registered in ISRCTN (ISRCTN16420548)
https://www.isrctn.com/ISRCTN16420548

Ethical approval: The study was approved by the Swedish Ethical Review Authority on 2021-08-11 (Dnr 2021-02855).

Word count: 4141

For peer review only

ABSTRACT

Introduction: Unhealthy lifestyle behaviours continue to be highly prevalent, including alcohol consumption, unhealthy diets, insufficient physical activity, and smoking. Despite evidence for the causal connection between modifiable lifestyle behaviours and non-communicable diseases, there is a lack of effective prevention interventions which have a large enough reach into the community to improve public health. Additionally, the common co-occurrence of multiple unhealthy lifestyle behaviours demands investigation of efforts which address more than single behaviours. One way forward is to determine how to best design digital multiple lifestyle behaviour interventions which can be offered to those who seek help online.

Methods and analysis: The effects of the components of a digital multiple lifestyle behaviour intervention on alcohol consumption, diet, physical activity, and smoking, will be estimated in a factorial randomised trial. The study population will be those seeking help online, recruited through search engines, social media, and lifestyle related websites. An adaptive design will be used to periodically make decisions to continue or stop recruitment, with simulations suggesting a final sample size between 1500 and 2500 participants. Multilevel regression models will be used to analyse behavioural outcomes collected at 2- and 4-months post-randomisation.

Ethics and dissemination: The study was approved by the Swedish Ethical Review Authority on 2021-08-11 (Dnr 2021-02855). Since participation is likely motivated by gaining access to novel support, the main concern is de-motivation and opportunity cost if the intervention is found to only exert small effects. Recruitment began on 2021-10-19, with an anticipated recruitment period of 12 months.

Registration: The trial was prospectively registered on 2021-10-05 (ISRCTN: ISRCTN16420548). The methods of this study, including the statistical analysis plan, was pre-registered prior to enrollment commenced on the Open Science Platform on 2021-10-19 (https://osf.io/xyj3p/).

ARTICLE SUMMARY

STRENGTHS AND LIMITATIONS OF THIS STUDY

- Pragmatic recruitment of individuals seeking help online to a factorial trial allow for dismantling of the effectiveness of the components which make up a digital multiple lifestyle behaviour intervention.
- An adaptive trial design reduces the risk of under- and over-recruitment of participants.
- Despite double blind procedures, research participation effects may affect self-reported outcomes and introduce bias.
- Single face-valid items used to measure mediators reduce participant burden but may limit the interpretation of findings.

For peer review only

INTRODUCTION

Behavioural risk factors, such as harmful alcohol consumption, unhealthy diets, insufficient physical activity, and smoking, contribute to about a third of global disability adjusted life years, and are leading causes of non-communicable diseases (NCDs), including cardiovascular disease, respiratory disease, cancer, and diabetes [1,2]. The World Health Organization has determined that reducing the prevalence of behavioural risk factors should be a priority in many societies to reduce the incidence of NCDs and disability adjusted life years [3]. It is therefore important that effective and scalable means of helping individuals to improve their lifestyle behaviours are established.

The Public Health Agency of Sweden's national public health survey from 2020 [4] (n = 16 947) reports data on lifestyle behaviours of Swedish citizens aged 16-84. According to the survey, 16% of respondents report hazardous or harmful alcohol consumption, 35% report being insufficiently physically active, 12% report smoking occasionally or daily, and 93% report eating less fruit and vegetables than recommended. Additionally, 52% of individuals report being obese or overweight. Unfortunately, with the exception of smoking, the prevalence rates of these behaviours have not decreased markedly over the past 10 years, with some increasing, witnessing of a lost decade for prevention efforts.

For prevention efforts to have an impact on the general population, they need to have extensive reach among those who may benefit. No single setting will be able to achieve this, e.g., only 1-5% of individuals visiting primary health care clinics in Sweden are given advice with respect to their lifestyle [5], despite many more in need of such advice. Unhealthy lifestyle behaviours also tend to cluster and interact [6,7], e.g. those who are overweight are more likely to be physically inactive, and excessive alcohol consumption may lead to weight gain. Risks from multiple unhealthy lifestyle behaviours may be multiplicative [8]; thus, it is of value to not only extend the reach of interventions, but to also investigate tools designed to support change of multiple lifestyle behaviours.

One way of reaching further into the community with a multiple lifestyle behaviour intervention is to offer digital support tools to those searching online for help. Studies evaluating digital interventions addressing multiple lifestyle behaviours have shown promising results [9–12]. However, the evidence of these types of interventions in more general populations is lacking, as the majority of studies have been conducted among university students, employees within specific fields, or patients with specific health conditions. In addition, behaviour interventions often consist of several components or modules, yet are commonly evaluated as a whole [13], leaving a paucity of evidence for the effects of the dismantled components. Increasing our understanding of the effects at the component level, in particular with respect to multiple lifestyles, may help move the field of behaviour interventions forward.

OBJECTIVES

This study aims to estimate the effects of the components of a digital intervention on multiple lifestyle behaviours (alcohol, physical activity, diet, and smoking) among individuals seeking help online. The objectives of the study are to:

1. Estimate the effects of a digital intervention's different components on individual lifestyle behaviours:
    a. Weekly alcohol consumption and number of episodes per month of heavy drinking.
    b. Average daily fruit and vegetable consumption.
    c. Weekly moderate to vigorous physical activity.
    d. Four-week point prevalence of smoking.

2. Estimate the degree to which the effects of the components are mediated through perceived importance, confidence, and know-how.

3. Detect interactions among lifestyle behaviour change, e.g., those who stop smoking may also reduce their alcohol consumption, and the degree to which this is moderated by the components of the intervention.

METHODS

A double blind factorial randomised trial [14] (6 factors with 2 levels each) will be employed to address the objectives of the study. A Bayesian group sequential design will be employed to periodically make decisions to continue or stop recruitment [15–17]. This protocol contains relevant items from the *Standard Protocol Items: Recommendations for Interventional Trials* (SPIRIT) [18]. The methods of this trial, including the statistical analysis plan, was pre-registered on the Open Science Platform prior to enrollment commenced (https://osf.io/xyj3p/).

STUDY SETTING, RECRUITMENT AND ELIGIBILITY

We will recruit individuals seeking information about lifestyle and behaviour change by advertising on Google, Bing, and Facebook (restricted to Sweden), as well as on websites which focus on lifestyle and behaviour change (e.g., livsstilsanalys.se). Individuals exposed to the advert will be advised to sign up to the study by sending a text message with a specific code to a dedicated phone number.

Within 10 minutes, individuals will receive a text message with a hyperlink that takes them to a web page with informed consent materials. Consent will be given by clicking on a button on the bottom of the page. All individuals giving informed consent will be asked to complete a baseline questionnaire, which will also assess eligibility for the trial (please see Appendix A). Individuals will be included in the trial if they fulfil at least one of five conditions:

- **Weekly alcohol consumption:** Consumed 10/15 (female/male) or more standard drinks of alcohol the past week. A standard drink of alcohol is in Sweden defined as 12 grams of pure alcohol.
- **Heavy episodic drinking:** Consumed 4/5 (female/male) or more standard drinks of alcohol on a single occasion at least once the past month.
- **Fruit and vegetables:** Consumed less than 500 grams of fruit and vegetables on average per day the past week.
- **Moderate to vigorous physical activity:** Spent less than 150 minutes on moderate to vigorous physical activity the past week.
- **Smoking:** Having smoked at least one cigarette the past week.

Individuals will be explicitly excluded if they do not fulfil any of the criteria or if they are less than 18 years of age. The trial information and intervention will be entirely in Swedish and delivered to participants' mobile phones, thus not comprehending Swedish well enough to sign up or not having access to a mobile phone will implicitly exclude individuals.

INTERVENTIONS

The digital intervention, which is called *Coach*, consists of six components which users access using their mobile phone, based on an intervention design we have used previously [19,20]. The intervention is designed around

For peer review only

social cognitive theories of behaviour change, with a focus on modifying environment, intention, and skills [21,22]. The intervention's components are intended to be used as a toolbox, allowing users to choose which parts of the intervention to interact with and tailor the support to their needs. The intervention materials can be accessed at participants' discretion over a 4-month period, and each Sunday afternoon participants will receive a text message with a link and a reminder to access the intervention materials.

The six components of the intervention are: (1) screening and feedback; (2) goalsetting and planning; (3) motivation; (4) skills and know-how; (5) mindfulness; and (6) self-authored text messages. These components will also represent factors in the factorial trial. Participants eligible for the trial will be randomly allocated to one of 64 factorial conditions, each condition representing a unique combination of the six components - which are either present or absent (2^6 = 64 conditions). They will remain in the same condition for the entirety of the 4-month intervention period. For a more detailed description of each component, including a full specification of each factorial condition, please see Appendix B.

For peer review only

OUTCOMES

MEASURES

Outcomes are listed here and subsequently explained. All questionnaires (baseline, 1-, 2- and 4-month follow-up) used in the trial can be found in Appendix A.

Primary outcome measures

- **Alcohol:** Weekly alcohol consumption; monthly frequency of heavy episodic drinking.
- **Diet:** Average daily consumption of fruit and vegetables.
- **Physical activity:** Weekly moderate to vigorous physical activity (MVPA).
- **Smoking:** Four-week point prevalence of smoking abstinence.

Secondary outcome measures

- Perceived stress.
- Weekly consumption of sugary drinks.
- Weekly consumption of candy and snacks.
- Body mass index (BMI).
- Weekly number of cigarettes smoked.
- Quality of life (QoL).

Mediation measures

- Importance of change.
- Confidence in one's ability to change.
- Knowledge of how to change.

PRIMARY AND SECONDARY OUTCOMES

Weekly alcohol consumption will be assessed by asking participants the number of standard drinks of alcohol they consumed last week (short term recall method [23]). Frequency of heavy episodic drinking will be assessed by asking participants how many times they have consumed more than 4/5 (female/male) standard drinks of alcohol on one occasion the past month. These two outcomes are both part of the proposed core outcome set for brief alcohol interventions [24–26].

Diet and physical activity will be measured utilising a questionnaire based on the previously published questionnaire by the National Board of Health and Welfare in Sweden [7], and was further modified to also include portion sizes. The consumption of fruit and vegetables will be measured using two questions concerning the number of portions (100 g) of fruit and vegetables (respectively) the participants ate on average per day during the past week. Sugary drinks consumption will be measured by a question regarding the number of units (33 cl) of sugary drinks participants consumed the past week, and candy and snacks will be measured using a single question regarding number of servings consumed last week. MVPA will be estimated by summing responses to two questions regarding the number of minutes spent on moderate and vigorous physical activity, respectively, during the past week.

Body mass index will be measured by asking participants to report their weight and height.

Four-week point prevalence of smoking abstinence (no cigarettes the past four weeks) will be asked as a binary question. This is a suggested measure by the Society of Research on Nicotine and Tobacco [27]. Participants who have smoked any cigarette the past four weeks will be asked for the number of cigarettes smoked the past week.

QoL will be measured using PROMIS Global 10 [28], both to estimate the degree to which intervention components effect QoL but also for health economic evaluations. Perceived stress will be assessed using the short form perceived stress scale (PSS-4) [29].

MEDIATION MEASURES

Participants will be asked to report on confidence, importance, and know-how; which are three psychosocial factors believed to be important markers of behaviour change [21,22,30–32]. To reduce participant burden, we will use single face-valid items, acknowledging the limitation of such measures.

PARTICIPANT TIMELINE AND FOLLOW-UPS

A trial participant timeline is presented in Figure 1. Intervention components (depending on allocation) will be made available to participants all at once and stay available to participants at their own discretion throughout the 4-month period (with weekly reminders). There are 3 follow-up stages: 1-, 2-, and 4-months post randomisation. All follow-ups will be initiated by sending text messages to participants with hyperlinks to questionnaires. The following additional attempts will be made to collect data:

1. A total of two text reminders will be sent two days apart to those who have not responded.

2. If there is no response to the mediator questions at the 1-month follow-up, then the questions will be sent in a text message and participants are asked to respond directly with a text.

3. If there is no response to the 2- and 4-month follow-ups, then we will call participants to collect responses for the primary outcome measures only. A maximum of 5 call attempts will be made.

For peer review only

INSERT FIGURE 1 HERE

Figure 1 - SPIRIT figure showing participant timeline throughout the study

ASSIGNMENT OF INTERVENTIONS

Randomisation will be fully automated and computerised. Block randomisation will be used to allocate participants to the 64 conditions (random block sizes of 64 and 128). Neither research personnel nor participants will be able to influence allocation.

Research personnel will be blind to allocation throughout the trial. All participants will have access to the intervention, albeit with different components, and they will not be made aware of the other available conditions and will therefore be blind to allocation.

PATIENT AND PARTICIPANT INVOLVEMENT STATEMENT

Outcome measures used in the trial are informed by national guidelines in Sweden, as well as those set by the WHO. Also, the Swedish National Board of Health and Welfare [7] have reported that research regarding multiple lifestyle behaviour change interventions is lacking. No patients or participants were involved in the planning of this trial or design of the intervention; however, both have been informed by our previous research involving individuals looking for help to change health related behaviours.

ANALYSIS

All analyses will be done keeping all participants in the groups to which they were randomised. Analyses will be done using both available data and imputation. Imputation will be done using multiple imputation with chained equations [33]. The implicit missing at random (MAR) assumption underlying this approach will be investigated by two attrition analyses: (1) if data is missing systematically then it may be the case that early responders (answering without reminders) differ from non-responders (requiring several attempts), and in extension that late responders are more alike non-responders. Therefore, one attrition analysis will regress primary outcomes against number of attempts to collect follow-up before a response was recorded; (2) we will further explore the MAR assumption by investigating if responders and non-responders are different with respect to baseline characteristics.

Groups will be contrasted using multilevel regression models with covariates for group by component interactions and participant level adaptive intercepts. Models of longitudinal data (primary outcomes and perceived stress) will include group by time by component interactions. We will explore pairwise interactions among components. Bayesian inference will be used to estimate the parameters of the models [34–36] (see Sample Size for priors). For each coefficient of interest, we will report the marginal posterior probability of effect, and the median will be used as a point estimate of the magnitude of the effect. We will also report on 50% and 95% compatibility intervals.

MODELS

PRIMARY AND SECONDARY OUTCOMES

Analyses of primary outcomes will be conducted among those fulfilling the respective criteria for inclusion at baseline, e.g., weekly alcohol consumption will be analysed among those who reported having consumed 10/15 (female/male) or more units of alcohol the past week. BMI, sugary drinks, candy/snacks, QoL, and

For peer review only - http://bmjopen.bmj.com/site/about/guidelines.xhtml

perceived stress will be analysed among all participants, and number of cigarettes smoked weekly among baseline smokers.

Weekly alcohol consumption, frequency of heavy episodic drinking per month, weekly intake of candy and snacks, number of sugary drinks per week, and cigarettes smoked per week are all count variables that are likely skewed and over dispersed. Therefore, these outcomes will be analysed using negative binomial regression. If found not to be over dispersed, we will consider using normal regression (possibly log transformed). Average intake of fruit and vegetables per day, MVPA minutes per week, BMI, QoL, and perceived stress will be analysed using normal regression (possibly log transformed). Point prevalence of smoking abstinence will be analysed using logistic regression.

All models will be adjusted for age, sex, and mediators (importance, confidence, and know-how) at baseline. Primary outcomes and perceived stress will be adjusted for their respective baseline values by virtue of time by component interactions, except for smoking prevalence which will be adjusted by the weekly number of cigarettes smoked at baseline. BMI, sugary drinks, and candy/snacks will be adjusted for baseline MVPA minutes per week and average intake of fruit and vegetables per day. Number of cigarettes smoked last week will be adjusted by its baseline value. QoL will be adjusted for perceived stress at baseline.

Effect modification will be explored in all models to assess if any of the baseline characteristics moderate the effects of the components of the intervention.

MEDIATOR OUTCOMES

Mediators will be explored using a causal inference framework [37–39], using Bayesian inference to estimate the natural direct effect and natural indirect effect (as per the definitions of Pearl [39]). We will report on the posterior distributions of these two estimates, as well as the proportion of the total effect which is accounted for by the natural indirect effect. Four models will be created for each primary outcome measure, three which investigate the mediating factors on their own, and a fourth which incorporates all mediators at once. If any baseline characteristics were found to moderate the effects in the primary analysis, then additional mediator models will be created to include these as moderators.

INTERACTIONS AMONG LIFESTYLE CHANGE

Outcome interactions, and determinants of such, will be investigated in an exploratory analysis. For instance, those who quit smoking may also be more likely to reduce their alcohol consumption, and this interaction may be moderated by baseline characteristics. In addition, we will investigate interactions between changes in perceived stress, QoL, and behaviour change. Models to detect such interactions will be explored and findings will be used to create hypotheses for future research.

SAMPLE SIZE

The trial will use a Bayesian group sequential design [15–17] to monitor recruitment with interim analyses planned for every 50 participants completing the 4-month follow-up. Each of the primary outcomes will be modelled according to the analysis plan (see Analysis), and coefficients for dummy variables representing presence/absence of each component at each follow-up interval will be assessed for effect, harm, and futility with respect to each outcome. We let $ß_{k,l,i}$ represent the regression coefficient for component *k*, at time *l*, for outcome *i*, and D all the data currently accumulated, then the target criteria will be:

- **Effect (fruit/veg. and physical activity):** $p(ß_{k,l,i} > 0 \mid D) > 97.5\%$ and $p(ß_{k,l,i} > 0.10 \mid D) > 50\%$
- **Harm (fruit/veg. and physical activity):** $p(ß_{k,l,i} < 0 \mid D) > 97.5\%$ and $p(ß_{k,l,i} < -0.10 \mid D) > 50\%$

For peer review only - http://bmjopen.bmj.com/site/about/guidelines.xhtml

- **Effect (alcohol and smoking):** $p(ß_{k,l,i} < 0 \mid D) > 97.5\%$ and $p(ß_{k,l,i} < -0.10 \mid D) > 50\%$
- **Harm (alcohol and smoking):** $p(ß_{k,l,i} > 0 \mid D) > 97.5\%$ and $p(ß_{k,l,i} > 0.10 \mid D) > 50\%$
- **Futility (all outcomes):** $p(-0.10 < ß_{k,l,i} < 0.10 \mid D) > 95\%$

Outcomes analysed using normal regression will be standardised when checking the above criteria. For the effect and harm criteria, we will use a standard normal prior for dummy covariates (mean = 0, sd = 1.0), and a slightly wider prior will be used for the futility criterion (mean = 0, sd = 2.0). The criteria should be viewed as targets, thus at each interim analysis we will evaluate each criterion and decide if we believe that recruitment should stop or continue. We will continue recruitment until one criterion is fulfilled for each component, for each outcome, at each follow-up interval. We will consider removing factors from the trial if the harm criteria are fulfilled. Note that we are estimating each component's effect on each outcome, thus we are not a-priori excluding any combination. If a component is ineffective with respect to a specific outcome, then this will be captured by the futility criteria, and will also be reported as a finding.

While the final sample size is not determined a-priori, we conducted a series of simulations with effect sizes at the minimal value of the above criteria (0.1 Cohen's d for fruit/veg and physical activity, 1.1 incidence rate ratios for alcohol, and 1.1 odds ratios for smoking). Simulations suggested that approximately 1500-2500 participants will be necessary to recruit. However, the criteria will decide, not the simulations. Despite having more conditions than in a traditional 2-arm trial (in this case 64 conditions), the factorial design is fully powered for each contrast [14]. This can be understood by observing that half the study population are given access to each individual component (see Supplementary Appendix Table 1 in Appendix B), thus the other half creates a contrast (a type of control).

Note that the Bayesian approach allows us to make unlimited looks at the data without worrying about multiplicities and error rates, as would be necessary using a frequentist approach [40]. Also, since no fixed effect size is pre-specified, we reduce the risk of stopping recruitment both too early and too late [17].

DISCUSSION

Maintaining a healthy diet and adequate physical exercise are proven ways to decrease the risk of many NCDs such as cancer and type II diabetes. More specifically, evidence suggests that the risk of many types of cancer is reduced by a diet which, among other things, includes vegetables and fruits and limits high-calorie foods and sugary drinks [41]. Smoking has been identified as the most prominent risk factor for developing many types of cancer, however, there are indications that more complex connections are in effect. For instance, alcohol consumption is a strong risk factor for cancer in and of itself, however, it has a synergetic relation with smoking in the context of developing certain types of cancer, meaning that a combination of these lifestyle behaviours amounts to bigger risks than their individual effects [42,43]. Research has provided strong evidence that risk factors for disease such as smoking, alcohol, physical inactivity and poor diet tend to have a clustered and co-occurring pattern in populations [44,45]. Swedish data shows a similar tendency, increasing the risk of poor health outcomes in the population and hence providing additional incitement for future studies to utilise a multi-behaviour approach. Furthermore, previous research concludes the need for future research to use a holistic approach, focusing on multiple and simultaneous interventions for behavioural change [10,44,46–49]

Two meta-analyses reported modest effects of multiple lifestyle intervention in non-clinical [47] and clinical populations [50], with various suggested reasons, including poor implementation. Some of the limitations of past efforts may be difficult to overcome with traditional face-to-face interventions, due to the large demand on staff and other resources. Only 4 of the 69 trials in one of the meta-analyses [47] investigated the use of interventions delivered via digital technology (e.g., email, text messages or websites). These trials were however limited by low power or engagement, targeted university students or young individuals, and had questionable external validity. All in all, despite the extended reach which digital lifestyle interventions may have, there is a lack of evidence for digital multiple lifestyle interventions targeting a more general population.

For peer review only - http://bmjopen.bmj.com/site/about/guidelines.xhtml

GENERALISABILITY AND LIMITATIONS

We have adopted a pragmatic recruitment strategy for this trial, using online channels, which closely mimics the way the intervention would be disseminated in a real-world context. The trial should therefore be viewed as estimating effectiveness of the intervention's components, rather than an efficacy. However, careful consideration should be taken due to the trial context creating expectations of and from participants [51,52], and those who take part in trials may be systematically different from those who do not. In addition, several limitations of the trial should be considered when interpreting findings.

The factorial design of this trial allows all participants to receive some support, even if some will receive a minimal number of components. Since conditions are unknown to participants we consider them blinded to allocation, which reduces the risk of bias [53,54]. This does not however protect entirely against social desirability bias, as those who are positive to the treatment received may want to support its dissemination by reporting more positive outcomes than actual [55], which may be less likely if fewer components of the intervention are received. Compensatory rivalry bias could exacerbate this issue [56]. We will ask questions with respect to participants' perceptions about the support received to support reasoning about the strength of these threats to validity.

Condition allocation may be revealed to research personnel when participants are called to collect follow-up data. This may be a source of bias, as non-blinded assessment of subjective measures have been found to bias estimates [57]. Deducing the exact allocation is however unlikely, and personnel are instructed to not ask about anything else than the follow-up data. Using phone calls is a strategy employed to reduce the risk of attrition bias, which we believe outweighs the risk of detection bias.

Finally, there are two methodological compromises which are important to address. First, we use single face-valid items for mediators to reduce participant burden, which means that any marked mediation effect should be carefully interpreted to relate to the full concept of importance, confidence, and know-how. Second, criteria for stopping enrolment are based on the analysis of individual components which does not consider interactions among components. While it would be advantageous to include criteria for interactions, it is not practical to do so as it would increase the expected sample size markedly.

ETHICS AND DISSEMINATION

The study was approved by the Swedish Ethical Review Authority on 2021-08-11 (Dnr 2021-02855). Participants are likely to have been motivated to sign up for the trial by the potential of receiving novel support, leading to a risk of opportunity cost if the intervention only exerts small effects on behaviour. However, considering that current prevention efforts seem to not be enough to reduce the prevalence of unhealthy lifestyle behaviours, and the potential effects and reach a digital multiple lifestyle behaviour intervention could have among those seeking help online, this risk was deemed acceptable.

Recruitment began in October 2021, and we anticipate that recruitment will last no more than 12 months. A final dataset will therefore be available in January 2023, and findings will be subsequently submitted for peer-review in open access journals.

For peer review only - http://bmjopen.bmj.com/site/about/guidelines.xhtml

STATEMENTS

AUTHORS CONTRIBUTIONS

Study objectives and outcomes were decided by MB, ML, PB, PH, and HH. MB and KÅ designed the trial and analysis plan. Intervention materials were conceptualised and developed by KÅ, JB, MB, OL, ML, PB, PH, and HH, based on an intervention design by MB. MB, KÅ and JB drafted the protocol, which was revised by ML, PB, PH, HH and OL – all authors contributed with intellectual content and approved the final version. JB, KÅ, and MB will be responsible for data collection and statistical analysis. All authors will be responsible for communication of findings from the trial.

FUNDING

This trial has been funded by The Swedish Cancer Society (Cancerfonden, 20 0883 Pj, PI: Dr. Marcus Bendtsen), and is an extension of the MoBILE research program which is funded by the Swedish Research Council for Health, Working Life and Welfare (Grant number 2018-01410; PI: Prof. Marie Löf).

COMPETING INTERESTS

MB and PB own a private company (Alexit AB) that develops and distributes lifestyle behaviour interventions for use in healthcare settings. Alexit AB had no part in funding or planning of this trial but is relied upon for a service to send text messages.

DATA AVAILABILITY STATEMENT

Data will be made available to researchers upon reasonable request, after approval of a research proposal and signing of data transfer agreements.

For peer review only

REFERENCES

1. World health organization. Fact sheet Noncommunicable diseases. 2018.

2. Stanaway JD, Afshin A, Gakidou E, Lim SS, Abate D, Abate KH, et al. Global, regional, and national comparative risk assessment of 84 behavioural, environmental and occupational, and metabolic risks or clusters of risks for 195 countries and territories, 1990–2017: a systematic analysis for the Global Burden of Disease Stu. The Lancet. 2018 Nov;392(10159):1923–94.

3. World Health Organization. Global action plan for the prevention and control of noncommunicable diseases 2013-2020. 2013.

4. Folkhälsomyndigheten. Bilaga 1 till ”Folkhälsans utveckling – Årsrapport 2021” - Resultat i tabellform [Internet]. 2021. Available from: https://www.folkhalsomyndigheten.se/globalassets/publicerat-material/publikationer/folkhalsan-arsrapport-2021/folkhalsans-utveckling-arsrapport-2021-bilaga-1-21014-1.pdf

5. Socialstyrelsen. Primärvårdens arbete med prevention och behandling av ohälsosamma levnadsvanor 2016. 2017.

6. Schuit AJ, Van Loon AJM, Tijhuis M, Ocké MC. Clustering of lifestyle risk factors in a general adult population. Prev Med. 2002;35(3):219–24.

7. Socialstyrelsen. Nationella riktlinjer för prevention och behandling vid ohälsosamma levnadsvanor. 2018.

8. Myint PK, Luben RN, Wareham NJ, Bingham SA, Khaw K. Combined effect of health behaviours and risk of first ever Norfolk cohort of European Prospective Investigation of Cancer (EPIC Norfolk): prospective population study. 2009;338(1):b349.

9. Deitz D, Cook RF, Hersch RK, Leaf S. Heart healthy online: an innovative approach to risk reduction in the workplace. J Occup Environ Med. 2014;56(5):547–53.

10. Schulz DN, Kremers SPJ, Vandelanotte C, Van Adrichem MJG, Schneider F, Candel MJJM, et al. Effects of a web-based tailored multiple-lifestyle intervention for adults: A two-year randomized controlled trial comparing sequential and simultaneous delivery modes. J Med Internet Res. 2014;16(1):e26.

11. Duncan MJ, Vandelanotte C, Trost SG, Rebar AL, Rogers N, Burton NW, et al. Balanced: a randomised trial examining the efficacy of two self-monitoring methods for an app-based multi-behaviour intervention to improve physical activity, sitting and sleep in adults. BMC Public Health. 2016 Dec;16(1):670.

12. A O, J B, A D, de Weerdt I, de Vries H, Oenema A, et al. Efficacy and use of an internet-delivered computer-tailored lifestyle intervention, targeting saturated fat intake, physical activity and smoking cessation: a randomized controlled trial. Ann Behav Med. 2008;35(2):125–35.

13. Pham Q, Wiljer D, Cafazzo JA. Beyond the Randomized Controlled Trial: A Review of Alternatives in mHealth Clinical Trial Methods. JMIR MHealth UHealth. 2016 Sep 9;4(3):e107.

14. Montgomery AA, Peters TJ, Little P. Design, analysis and presentation of factorial randomised controlled trials. BMC Med Res Methodol. 2003 Dec 24;3(1):26.

15. Gsponer T, Gerber F, Bornkamp B, Ohlssen D, Vandemeulebroecke M, Schmidli H. A practical guide to Bayesian group sequential designs. Pharm Stat. 2014;13(1):71–80.

16. Berry DA. Bayesian clinical trials. Nat Rev Drug Discov. 2006 Jan;5(1):27–36.

17. Bendtsen M. The P Value Line Dance: When Does the Music Stop? J Med Internet Res. 2020;22(8):e21345.

18. Chan A-W, Tetzlaff JM, Altman DG, Laupacis A, Gøtzsche PC, Krleža-Jerić K, et al. SPIRIT 2013 Statement: Defining Standard Protocol Items for Clinical Trials. Ann Intern Med. 2013 Feb 5;158(3):200.

19. Bendtsen M, McCambridge J. Reducing Alcohol Consumption Among Risky Drinkers in the General Population of Sweden Using an Interactive Mobile Health Intervention: Protocol for a Randomized Controlled Trial. JMIR Res Protoc. 2019;8(4):e13119.

20. Åsberg K, Lundgren O, Henriksson H, Henriksson P, Bendtsen P, Löf M, et al. Multiple lifestyle behaviour mHealth intervention targeting Swedish college and university students: protocol for the *Buddy* randomised factorial trial. BMJ Open. 2021 Dec;11(12):e051044.

21. Fishbein M, Triandis HC, Kanfer FH, Becker M, Middlestadt SE, Eichler A. Factors influencing behaviour and behaviour change. In: Handbook of Health Psychology. Psychology Press Taylor & Francis Group; 2001. p. 3–17.

22. Conner M, Norman P. Predicting Health Behavior: Research and Practice with Social Cognition Models. 2005.

23. Rehm J. Measuring Quantity, Frequency, and Volume of Drinking. Alcohol Clin Exp Res. 1998;22(s2):4s–14s.

24. Shorter GW, Heather N, Bray JW, Giles EL, Holloway A, Barbosa C, et al. The 'Outcome Reporting in Brief Intervention Trials: Alcohol' (ORBITAL) framework: protocol to determine a core outcome set for efficacy and effectiveness trials of alcohol screening and brief intervention. Trials. 2017 Dec;18(1):611.

For peer review only

25. Shorter GW, Bray JW, Giles EL, O'Donnell AJ, Berman AH, Holloway A, et al. The Variability of Outcomes Used in Efficacy and Effectiveness Trials of Alcohol Brief Interventions: A Systematic Review. J Stud Alcohol Drugs. 2019 May;80(3):286–98.

26. Bendtsen M, Garnett C, Toner P, Shorter GW. The Effect of Question Order on Outcomes in the Core Outcome Set for Brief Alcohol Interventions Among Online Help-Seekers: Protocol for a Factorial Randomized Trial. JMIR Res Protoc. 2020 Nov 26;9(11):e24175.

27. SRNT Subcommittee on Biochemical Verification. Biochemical verification of tobacco use and cessation. Nicotine Tob Res. 2002 May;4(2):149–59.

28. Hays RD, Bjorner JB, Revicki DA, Spritzer KL, Cella D. Development of physical and mental health summary scores from the patient-reported outcomes measurement information system (PROMIS) global items. Qual Life Res. 2009 Sep;18(7):873–80.

29. Vallejo MA, Vallejo-Slocker L, Fernández-Abascal EG, Mañanes G. Determining Factors for Stress Perception Assessed with the Perceived Stress Scale (PSS-4) in Spanish and Other European Samples. Front Psychol. 2018 Jan 26;9.

30. Ajzen I. The theory of planned behavior. Organ Behav Hum Decis Process. 1991;50(2):179–211.

31. Bandura A. Self Efficacy: the exercise of control. Worth Publishers; 1997.

32. Rogers R. Cognitive and physiological processes in fear appeals and attitude change: A revised theory of protection motivation. In: Social Psychophysiological: A Sourcebook. 1983.

33. White IR, Royston P, Wood AM. Multiple imputation using chained equations: Issues and guidance for practice. Stat Med. 2011;30(4):377–99.

34. Bendtsen M. A Gentle Introduction to the Comparison Between Null Hypothesis Testing and Bayesian Analysis: Reanalysis of Two Randomized Controlled Trials. J Med Internet Res. 2018;20(10):e10873.

35. Bendtsen M. Electronic Screening for Alcohol Use and Brief Intervention by Email for University Students: Reanalysis of Findings From a Randomized Controlled Trial Using a Bayesian Framework. J Med Internet Res. 2019;21(11):e14419.

36. Bendtsen M. An Electronic Screening and Brief Intervention for Hazardous and Harmful Drinking Among Swedish University Students: Reanalysis of Findings From a Randomized Controlled Trial Using a Bayesian Framework. J Med Internet Res. 2019;21(12):e14420.

37. Imai K, Keele L, Tingley D. A General Approach to Causal Mediation Analysis. Psychol Methods. 2010;15(4):309–34.

38. Pearl J. Causality. Causality: Models, Reasoning, and Inference, Second Edition. Cambridge: Cambridge University Press; 2009.

39. Pearl J. Interpretation and identification of causal mediation. Psychol Methods. 2014;19(4):459–81.

40. Harrell F. Continuous Learning from Data: No Multiplicities from Computing and Using Bayesian Posterior Probabilities as Often as Desired [Internet]. 2020 [cited 2020 May 10]. Available from: https://www.fharrell.com/post/bayes-seq/

41. Norat T, Scoccianti C, Boutron-Ruault M-C, Anderson A, Berrino F, Cecchini M, et al. European Code against Cancer 4th Edition: Diet and cancer. Cancer Epidemiol. 2015 Dec;39:S56–66.

42. Leon ME, Peruga A, McNeill A, Kralikova E, Guha N, Minozzi S, et al. European Code against Cancer, 4th Edition: Tobacco and cancer. Cancer Epidemiol. 2015 Dec;39:S20–33.

43. Scoccianti C, Cecchini M, Anderson AS, Berrino F, Boutron-Ruault M-C, Espina C, et al. European Code against Cancer 4th Edition: Alcohol drinking and cancer. Cancer Epidemiol. 2016 Dec;45:181–8.

For peer review only

44. Noble N, Paul C, Turon H, Oldmeadow C. Which modifiable health risk behaviours are related? A systematic review of the clustering of Smoking, Nutrition, Alcohol and Physical activity ('SNAP') health risk factors. Prev Med. 2015 Dec;81:16–41.

45. Berrigan D, Dodd K, Troiano RP, Krebs-Smith SM, Barbash RB. Patterns of health behavior in U.S. adults. Prev Med. 2003 May;36(5):615–23.

46. Bendtsen M, Bendtsen P, Henriksson H, Henriksson P, Müssener U, Thomas K, et al. The Mobile Health Multiple Lifestyle Behavior Interventions Across the Lifespan (MoBILE) Research Program: Protocol for Development, Evaluation, and Implementation. JMIR Res Protoc. 2020;9(4):e14894.

47. Meader N, King K, Wright K, Graham HM, Petticrew M, Power C, et al. Multiple Risk Behavior Interventions: Meta-analyses of RCTs. Am J Prev Med. 2017;53(1):e19–30.

48. Prochaska JJ, Spring B, Nigg CR. Multiple health behavior change research: An introduction and overview. Prev Med. 2008;46(3):181–8.

49. De Vries H, Kremers S, Smeets T, Reubsaet A. Clustering of diet, physical activity and smoking and a general willingness to change. Psychol Health. 2008;23(3):265–78.

50. Alageel S, Gulliford MC, McDermott L, Wright AJ. Multiple health behaviour change interventions for primary prevention of cardiovascular disease in primary care: systematic review and meta-analysis. BMJ Open. 2017 Jun;7(6):e015375.

51. McCambridge J, Kypri K, Elbourne D. Research participation effects: a skeleton in the methodological cupboard. J Clin Epidemiol. 2014 Aug;67(8):845–9.

52. McCambridge J, Witton J, Elbourne DR. Systematic review of the Hawthorne effect: New concepts are needed to study research participation effects. J Clin Epidemiol. 2014 Mar;67(3):267–77.

53. Higgins JPT, Altman DG, Gotzsche PC, Juni P, Moher D, Oxman AD, et al. The Cochrane Collaboration's tool for assessing risk of bias in randomised trials. BMJ. 2011 Oct 18;343(oct18 2):d5928–d5928.

54. Bendtsen M, McCambridge J, Åsberg K, Bendtsen P. Text Messaging Interventions for Reducing Alcohol Consumption Among risky drinkers: Systematic Review and Meta-Analysis. Addiction. 2020;

55. Miles LM, Elbourne D, Farmer A, Gulliford M, Locock L, McCambridge J, et al. Bias due to MEasurement Reactions In Trials to improve health (MERIT): protocol for research to develop MRC guidance. Trials. 2018 Dec 26;19(1):653.

56. McCambridge J. From question-behaviour effects in trials to the social psychology of research participation. Psychol Health. 2015 Jan 2;30(1):72–84.

57. Hrobjartsson A, Thomsen ASS, Emanuelsson F, Tendal B, Hilden J, Boutron I, et al. Observer bias in randomised clinical trials with binary outcomes: systematic review of trials with both blinded and non-blinded outcome assessors. BMJ. 2012 Feb 27;344(feb27 2):e1119–e1119.

FIGURE LEGENDS

Figure 1 - SPIRIT figure depicting participant timeline.

For peer review only

	STUDY PERIOD					
	**Enrolment**	**Allocation**	**Post-allocation**			**Close-out**
**TIMEPOINT**	**0**	**0**	**0**	**1 month**	**2 months**	**4 months**
**ENROLMENT:**						
**Informed consent**	X					
**Eligibility screen**	X					
**Allocation**		X				
**INTERVENTIONS:**						
Digital intervention (factorial design)		X	◆———	———	———	———◆
**ASSESSMENTS:**						
Baseline questionnaire	X					
Mediator questionnaire	X			X	X	X
Lifestyle outcomes questionnaire					X	X
Perceived stress	X				X	X
QoL						X
Participant experience						X

For peer review only

APPENDIX A – QUESTIONNAIRES

Note: Participants are reminded of the definition of a standard drink of alcohol by graphical means, as well as given visual cues for what constitutes a portion of fruit and vegetables.

BASELINE QUESTIONNAIRE

1. Sex:
    a. Female
    b. Male

2. Age (numerical measure)

3. How many standard drinks of alcohol did you consume last week? (numerical measure)

4. How often, during the past month, have you consumed four/five (female/male) or more standard drinks of alcohol on one occasion? (numerical measure)

5. How many cigarettes did you smoke last week? (numerical measure)

6. How much time in total did you spend on moderate physical activity (e.g. bicycling or walking for transport or leisure) **last week**?
    a. 0
    b. Less than 30 minutes
    c. 30-60 minutes
    d. 1 hours
    e. 1.5 hours
    f. 2 hours
    g. 2.5 hours
    h. 3 hours
    i. 3.5 hours (i.e. 30 minutes per day)
    j. 4 hours
    k. 5 hours
    l. 6 hours
    m. 7 hours (i.e. 1 hour per day)
    n. 10.5 hours (i.e. 1.5 hours per day)
    o. 14 hours (i.e. 2 hours per day)

7. How much time in total did you spend on vigorous physical activity (i.e. producing increases in breathing or heart rate), for instance running, aerobics, etc. **last week?**
    a. 0
    b. Less than 30 minutes
    c. 30-60 minutes
    d. 1 hours
    e. 1.5 hours
    f. 2 hours
    g. 2.5 hours
    h. 3 hours
    i. 3.5 hours (i.e. 30 minutes per day)

For peer review only

j. 4 hours
k. 5 hours
l. 6 hours
m. 7 hours (i.e. 1 hour per day)
n. 10.5 hours (i.e. 1.5 hours per day)
o. 14 hours (i.e. 2 hours per day)

8. How many 100g portions (equivalent to an average sized banana or one large apple) of fruit did you consume **last week**?
    a. 0
    b. 1-2 portions **per week**
    c. 3-4 portions **per week**
    d. 5-6 portion **per week**
    e. 1.0 portion **per day**
    f. 1.5 portions **per day**
    g. 2.0 portions **per day**
    h. 2.5 portions **per day**
    i. 3.0 portions **per day or more**

9. How many 100 g portions (equivalent to an average handful) of vegetables did you consume **last week**?
    a. 0
    b. 1-2 portions **per week**
    c. 3-4 portions **per week**
    d. 5-6 portion **per week**
    e. 1.0 portion **per day**
    f. 1.5 portions **per day**
    g. 2.0 portions **per day**
    h. 2.5 portions **per day**
    i. 3.0 portions **per day or more**

10. In the last month, how often have you felt that you were unable to control the important things in your life?
    a. Never
    b. Almost never
    c. Sometimes
    d. Fairly often
    e. Very often

11. In the last month, how often have you felt confident about your ability to handle your personal problems?
    a. Never
    b. Almost never
    c. Sometimes
    d. Fairly often
    e. Very often

12. In the last month, how often have you felt that things were going your way?
    a. Never
    b. Almost never

For peer review only

c. Sometimes
d. Fairly often
e. Very often

13. In the last month, how often have you felt difficulties were piling up so high that you could not overcome them?
    a. Never
    b. Almost never
    c. Sometimes
    d. Fairly often
    e. Very often

14. How important is it for you to improve your lifestyle behaviours? (10-point scale ranging from 1 = "Not important" to 10 = "Very important")

15. How confident are you that you will be able to improve your lifestyle behaviours? (10-point scale ranging from 1 = "Not at all" to 10 = "Very confident")

16. To what degree do you have the know-how and strategies to improve your lifestyle behaviours? (10-point scale ranging from 1 = "Not at all" to 10 = "Very high degree")

1-MONTH FOLLOW-UP (MEDIATORS ONLY) QUESTIONNAIRE

1. How important is it for you to improve your lifestyle behaviours? (10-point scale ranging from 1 = "Not important" to 10 = "Very important")

2. How confident are you that you will be able to improve your lifestyle behaviours? (10-point scale ranging from 1 = "Not at all" to 10 = "Very confident")

3. To what degree do you have the know-how and strategies to improve your lifestyle behaviours? (10-point scale ranging from 1 = "Not at all" to 10 = "Very high degree")

2- AND 4-MONTH FOLLOW-UP QUESTIONNAIRE

1. How many standard drinks of alcohol did you consume last week? (numerical measure)

2. How often, during the past month, have you consumed four/five (female/male) or more standard drinks of alcohol on one occasion? (numerical measure)

3. Have you smoked any cigarettes the past four weeks?
    a. Yes
    b. No

4. (Smokers only) How many cigarettes did you smoke last week? (numerical measure)

5. How much time in total did you spend on moderate physical activity (e.g. bicycling or walking for transport or leisure) **last week**?
    a. 0
    b. Less than 30 minutes
    c. 30-60 minutes
    d. 1 hours

   e. 1.5 hours
   f. 2 hours
   g. 2.5 hours
   h. 3 hours
   i. 3.5 hours (i.e. 30 minutes per day)
   j. 4 hours
   k. 5 hours
   l. 6 hours
   m. 7 hours (i.e. 1 hour per day)
   n. 10.5 hours (i.e. 1.5 hours per day)
   o. 14 hours (i.e. 2 hours per day)

6. How much time in total did you spend on vigorous physical activity (i.e. producing increases in breathing or heart rate), for instance running, aerobics, etc. **last week?**
   a. 0
   b. Less than 30 minutes
   c. 30-60 minutes
   d. 1 hours
   e. 1.5 hours
   f. 2 hours
   g. 2.5 hours
   h. 3 hours
   i. 3.5 hours (i.e. 30 minutes per day)
   j. 4 hours
   k. 5 hours
   l. 6 hours
   m. 7 hours (i.e. 1 hour per day)
   n. 10.5 hours (i.e. 1.5 hours per day)
   o. 14 hours (i.e. 2 hours per day)

7. How many 100g portions (equivalent to an average sized banana or one large apple) of fruit did you consume **last week**?
   a. 0
   b. 1-2 portions **per week**
   c. 3-4 portions **per week**
   d. 5-6 portion **per week**
   e. 1.0 portion **per day**
   f. 1.5 portions **per day**
   g. 2.0 portions **per day**
   h. 2.5 portions **per day**
   i. 3.0 portions **per day or more**

8. How many 100 g portions (equivalent to an average handful) of vegetables did you consume **last week**?
   a. 0
   b. 1-2 portions **per week**
   c. 3-4 portions **per week**
   d. 5-6 portion **per week**
   e. 1.0 portion **per day**
   f. 1.5 portions **per day**

For peer review only

g. 2.0 portions **per day**
h. 2.5 portions **per day**
i. 3.0 portions **per day or more**

9. How many cans (33 cl, one standard can) of sugary drinks (e.g. soft/fizzy drinks, “energy drinks”) did you consume **last week**?
    a. 0 cans
    b. 1 can **per week**
    c. 2-3 cans **per week**
    d. 4-6 cans **per week**
    e. 1 can **per day**
    f. 1.5 cans **per day**
    g. 2.0 cans **per day**
    h. 2.5 cans **per day**
    i. 3.0 cans **per day or more**

10. How many portions of sweets, chocolate, pastry (e.g. buns, muffins, biscuits), ice cream and salty snacks (e.g. crisps, nuts, cheese doodles) did you eat **last week**? *One portion is 50 g sweets (9 pieces), 40 g chocolate (6 pieces/squares), 1 bun, 2 dl (scoops) of ice cream or 2 dl snacks (40 g).*

    a. 0 portions
    b. 1 portion **per week**
    c. 2-3 portions **per week**
    d. 4-6 portions **per week**
    e. 1 portion **per day**
    f. 1.5 portions **per day**
    g. 2.0 portions **per day**
    h. 2.5 portions **per day**
    i. 3.0 portions **per day**
    j. 3.5 portions **per day**
    k. 4.0 portions **per day or more**

11. How tall are you? (numerical measure)

12. What is your current body weight? (numerical measure)

13. In the last month, how often have you felt that you were unable to control the important things in your life?
    a. Never
    b. Almost never
    c. Sometimes
    d. Fairly often
    e. Very often

14. In the last month, how often have you felt confident about your ability to handle your personal problems?
    a. Never
    b. Almost never
    c. Sometimes

For peer review only

d. Fairly often
e. Very often

15. In the last month, how often have you felt that things were going your way?
    a. Never
    b. Almost never
    c. Sometimes
    d. Fairly often
    e. Very often

16. In the last month, how often have you felt difficulties were piling up so high that you could not overcome them?
    a. Never
    b. Almost never
    c. Sometimes
    d. Fairly often
    e. Very often

17. How important is it for you to improve or maintain healthy lifestyle behaviours? (10-point scale ranging from 1 = "Not important" to 10 = "Very important")

18. How confident are you that you will be able to improve or maintain healthy lifestyle behaviours? (10-point scale ranging from 1 = "Not at all" to 10 = "Very confident")

19. To what degree do you have the know-how and strategies to improve or maintain healthy lifestyle behaviours? (10-point scale ranging from 1 = "Not at all" to 10 = "Very high degree")

4-MONTH FOLLOW-UP ONLY

1. Overall, how well do you believe that the support given to you suited your needs?
    a. I feel like I did not receive any support at all
    b. I feel like I received some support, but it did not suit my needs
    c. I feel like I received some support, and it did suit my needs
    d. I feel like I received all the support that I needed

2. (If a or b to question 2): You have responded that you did not receive adequate support, what did you do instead?
    a. I decided to find other ways to help me change my lifestyle
    b. I decided to not make any change to my lifestyle
    c. Other (please comment)

3. Please leave a comment describing your needs and how the support did or did not address them (Free-text).

4. Do you believe that the support given to you would be helpful for other individuals that want to change their lifestyle? (1 = "Not very helpful" to 5 = "Very helpful")

5. Would you recommend the support you were given to a friend who expresses a wish to change their lifestyle?
    a. Yes

For peer review only

   b. No
   c. I do not know

6. If you were to continue using the support, for how much longer would you want to use it?
   a. I would use it for one to two more months
   b. I would use it for three to six more months
   c. I would use it for more than six months
   d. I would not use it any more
   e. I do not know

7. In general, would you say your health is: (Poor, Fair, Good, Very good, Excellent)

8. In general, would you say your quality of life is: (Poor, Fair, Good, Very good, Excellent)

9. In general, how would you rate your physical health: (Poor, Fair, Good, Very good, Excellent)

10. In general, how would you rate your mental health, including your mood and your ability to think? (Poor, Fair, Good, Very good, Excellent)

11. In general, how would you rate your satisfaction with your social activities and relationships? (Poor, Fair, Good, Very good, Excellent)

12. In general, please rate how well you carry out your usual social activities. This includes activities at home, at work and in your community, and responsibilities as a parent, child, spouse, employee, friend, etc.: (Poor, Fair, Good, Very good, Excellent)

13. To what extent are you able to carry out your everyday physical activities such as walking, climbing stairs, carrying groceries, or moving a chair?
   a. Not at all
   b. A little
   c. Moderately
   d. Mostly
   e. Completely

14. In the past 7 days, how often have you been bothered by emotional problems such as feeling anxious depressed or irritable?
   a. Always
   b. Often
   c. Sometimes
   d. Rarely
   e. Never

15. In the past 7 days, how would you rate your fatigue on average?
   a. Very severe
   b. Severe
   c. Moderate
   d. Mild
   e. None

For peer review only

16. In the past 7 days, how would you rate your pain on average (where 0 is No Pain, and 10 is Worst Pain Imaginable)?

For peer review only

APPENDIX B – INTERVENTION DESCRIPTION AND FACTORIAL CONDITIONS

This appendix describes the content of the *Coach* intervention, which is based on an intervention design we have used previously [1,2]. The intervention targets alcohol, diet, physical activity, and smoking. The factorial conditions of the trial are also explained in detail in this appendix.

The intervention was developed with inspiration from the first four steps of the Intervention Mapping (IM) approach [3]. The intervention is based on social cognitive models for behaviour change where environment, intentions and skills often are highlighted as important for change [4,5]. Therefore, we identified and designed components which intended to affect these factors. This was based on our previous research in Sweden [1,6–12] and the research literature more widely (see specific descriptions below). The logic model in Supplementary Appendix Figure 1 gives an overview of the reasoning behind the intervention, including outcomes and potential short-, mid- and long-term impact.

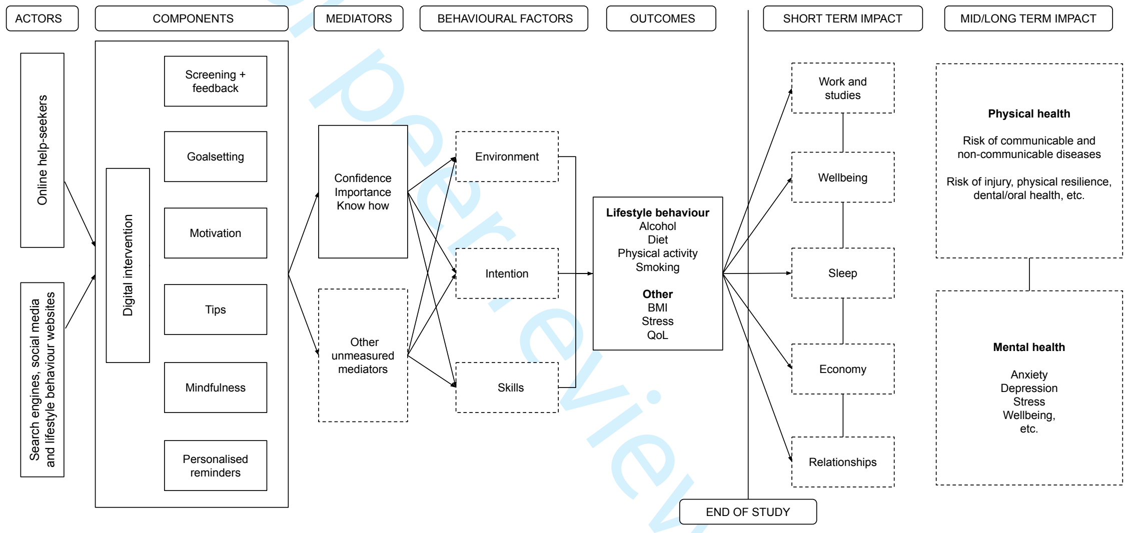

Supplementary Appendix Figure 1 - Logic model showing actors, intervention components, mediators, behavioural factors, outcomes, and short-, mid- and long-term impacts

INTERVENTION DESIGN AND COMPONENTS

The intervention is intended to be used as a toolbox, enabling users to decide which intervention content they want to interact with and when. As can be seen in the screenshot in Supplementary Appendix Figure 2, the design of the intervention allows for each component to be presented to participants in a menu, allowing easy addition and removal of components based on factorial condition. Follows does a description of each component, using the BCTTv1 93-item taxonomy [13] to specify techniques included when appropriate.

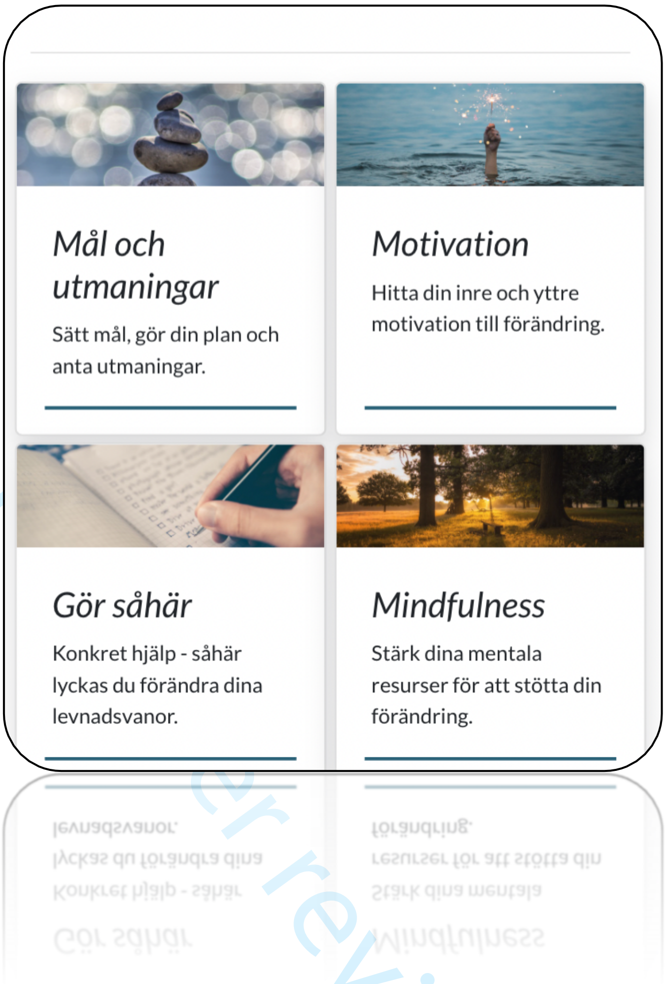

Supplementary Appendix Figure 2 - A screenshot of the digital intervention showing the main menu

COMPONENT 1: SCREENING AND FEEDBACK

The first component consists of screening and feedback. Every Sunday afternoon, participants will receive a text message with a hyperlink. When pressing the link, participants will be asked to respond to a questionnaire regarding their current lifestyle behaviours, after which they are shown feedback on their current behaviour in contrast to national guidelines (see screenshots in Supplementary Appendix Figure 3). They will subsequently be given access to the rest of the components appropriate for their randomised allocation. Self-monitoring has been shown to be a potentially effective strategy for reducing excessive alcohol consumption [14–17] and to promote healthy eating and physical activity [18,19]. When this component is absent, participants will not be asked to respond to the screening questionnaire but will instead be shown national guidelines without any feedback. BCTs used: Discrepancy between current behaviour and goal (BCT 1.6), Feedback on behaviour (BCT 2.2), Self-monitoring of behaviour (BCT 2.3), and Social comparison (BCT 6.2).

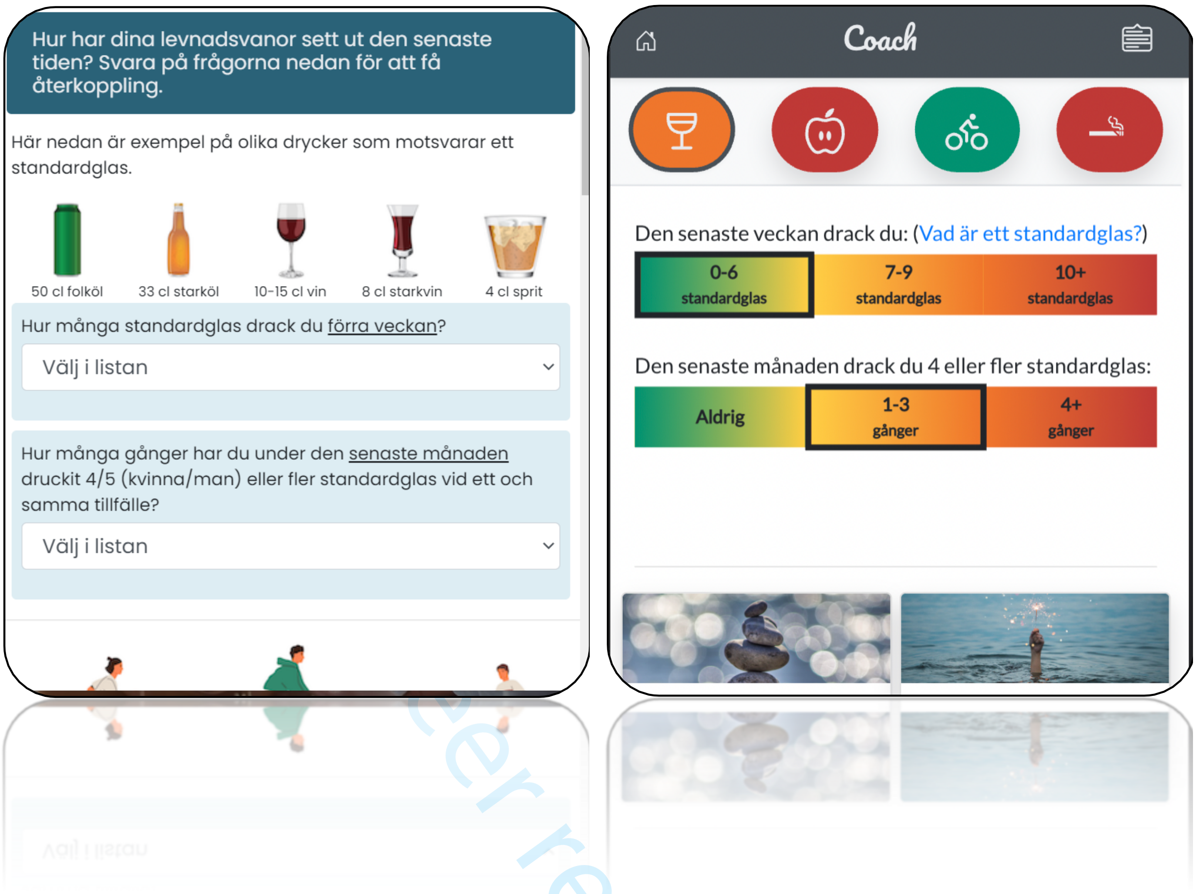

Supplementary Appendix Figure 3 – Screenshots of the digital intervention showing screening and feedback based on national guidelines

COMPONENT 2: GOALSETTING AND PLANNING

The second component supports enhanced self-regulatory capacity and skills via goalsetting and planning. This includes setting goals for future behaviour, preparing for triggers, and accepting both custom and ready-made challenges. Intervention content designed around goalsetting, action planning, practicing behaviour, and habit formation have, amongst other planning related activities, been shown to be important among effective lifestyle interventions [18,20–25]. Participants will be reminded of the goals that they have set, including any challenges they have accepted, via text message prompts throughout the week (up to 4 messages). BCTs used: Goal setting (behaviour) (BCT 1.1), Problem solving (BCT 1.2), Action planning (BCT 1.4), Prompts/cues (BCT 7.1), Behaviour practice/rehearsal (BCT 8.1), Behaviour substitution (BCT 8.2), Habit formation (BCT 8.3), Graded tasks (BCT 8.7).

COMPONENT 3: MOTIVATION

The third component aims to increase users' awareness of their own motivation, prompt commitment, and boost motivation. This is supported via texts, videos and exercises relating to health, economics, and motivation awareness. Digital behaviour change interventions have been shown to have the capacity to increase self-efficacy, however, there is lack of consensus across reviews with regards to which content works to facilitate an increase of self-efficacy [26]. The component will also allow participants to sign up for text messages with motivational content sent to them throughout the week. Participants choose which behaviours

For peer review only

they wish to have messages for, with a maximum of 8-10 messages per week. The content of the messages has been derived from previously developed and evaluated interventions [6–12]. BCTs used: Information about health consequences (BCT 5.1), Credible source (9.1), Pros and cons (BCT 9.2), Comparative imagining of future outcomes (BCT 9.3).

COMPONENT 4: SKILLS AND KNOW-HOW

The fourth component aims to increase user's skills and know-how of how to make lasting behavioural changes. This will include concrete tips on how to initiate and maintain change in everyday life. For instance, participants are given strategies they can employ when going to parties where alcohol is served, or how to introduce vegetables to their meals. As with the third component, participants will be able to sign up for text messages with tips sent to them throughout the week (maximum 8-10 per week) – the content of which has also been derived from previously developed and evaluated interventions [6–12]. BCTs used: Social support (unspecified) (BCT 3.1), Instructions on how to perform a behaviour (BCT 4.1), Self-incentive (BCT 10.7), and Self-reward (BCT 10.9).

COMPONENT 5: MINDFULNESS

The fifth component aims to increase users' awareness of their own lived experience and strengthen their capacity for a non-reactive, compassionate, and less stressful way of being in the world. The practices thus help participants to build the mental resources needed for behaviour change. A set of mindfulness exercises, including guided meditations, will be available in the component. The exercises are based on previous research, and are considered evidence-based methods to improve the mental well-being of clinical populations, while effects in non-clinical settings and behaviour change are less studied [27–31].

COMPONENT 6: SELF-COMPOSED TEXT MESSAGES

The sixth component consists of self-composed text messages sent to participants throughout the week. Participants will be allowed to author up to three messages to themselves and have them sent at specified intervals. For instance, a participant can write a message about their commitment to increase their physical activity and decide to have it sent to them every Monday and Wednesday at 5pm. This type of activity seems generally under-studied in the literature, but has shown preliminary interesting results in an ongoing trial [32].

For peer review only

FACTORIAL ALLOCATIONS

Using P to represent *present* and A to represent *absent*, the 64 factorial conditions are presented in Supplementary Appendix Table 1. As is evident from the table, each component will be available to half the study population, allowing for contrasts between *present* and *absent* to be fully powered by the sample. However, as one is estimating the effects of individual components, effect sizes may be smaller than when contrasting the full intervention versus a control, which may increase the sample size required.

Supplementary Appendix Table 1 - Combination of components in 64 factorial conditions (P = present , A = absent)

Conditions	Component 1	Component 2	Component 3	Component 4	Component 5	Component 6
1	P	P	P	P	P	P
2	P	P	P	P	P	A
3	P	P	P	P	A	P
4	P	P	P	P	A	A
5	P	P	P	A	P	P
6	P	P	P	A	P	A
7	P	P	P	A	A	P
8	P	P	P	A	A	A
9	P	P	A	P	P	P
10	P	P	A	P	P	A
11	P	P	A	P	A	P
12	P	P	A	P	A	A
13	P	P	A	A	P	P
14	P	P	A	A	P	A
15	P	P	A	A	A	P
16	P	P	A	A	A	A
17	P	A	P	P	P	P
18	P	A	P	P	P	A
19	P	A	P	P	A	P
20	P	A	P	P	A	A
21	P	A	P	A	P	P
22	P	A	P	A	P	A
23	P	A	P	A	A	P
24	P	A	P	A	A	A
25	P	A	A	P	P	P
26	P	A	A	P	P	A
27	P	A	A	P	A	P
28	P	A	A	P	A	A
29	P	A	A	A	P	P
30	P	A	A	A	P	A
31	P	A	A	A	A	P
32	P	A	A	A	A	A
33	A	P	P	P	P	P

For peer review only

34	A	P	P	P	P	A
35	A	P	P	P	A	P
36	A	P	P	P	A	A
37	A	P	P	A	P	P
38	A	P	P	A	P	A
39	A	P	P	A	A	P
40	A	P	P	A	A	A
41	A	P	A	P	P	P
42	A	P	A	P	P	A
43	A	P	A	P	A	P
44	A	P	A	P	A	A
45	A	P	A	A	P	P
46	A	P	A	A	P	A
47	A	P	A	A	A	P
48	A	P	A	A	A	A
49	A	A	P	P	P	P
50	A	A	P	P	P	A
51	A	A	P	P	A	P
52	A	A	P	P	A	A
53	A	A	P	A	P	P
54	A	A	P	A	P	A
55	A	A	P	A	A	P
56	A	A	P	A	A	A
57	A	A	A	P	P	P
58	A	A	A	P	P	A
59	A	A	A	P	A	P
60	A	A	A	P	A	A
61	A	A	A	A	P	P
62	A	A	A	A	P	A
63	A	A	A	A	A	P
64	A	A	A	A	A	A

REFERENCES

1. Bendtsen M, McCambridge J. Reducing alcohol consumption among risky drinkers in the general population of Sweden using an interactive mobile health intervention: protocol for a randomized controlled trial. JMIR Research Protocols. 2019;8(4):e13119.

2. Åsberg K, Lundgren O, Henriksson H, Henriksson P, Bendtsen P, Löf M, et al. Multiple lifestyle behaviour mHealth intervention targeting Swedish college and university students: protocol for the *Buddy* randomised factorial trial. BMJ Open. 2021 Dec;11(12):e051044.

3. Bartholomew Eldredge LK. Planning health promotion programs: an intervention mapping approach. Fourth edition. San Francisco, CA: Jossey-Bass & Pfeiffer Imprints, Wiley; 2016. 1 p.

For peer review only

4. Fishbein M, Triandis HC, Kanfer FH, Becker M, Middlestadt SE, Eichler A. Factors influencing behaviour and behaviour change. In: Handbook of Health Psychology. Psychology Press Taylor & Francis Group; 2001. p. 3–17.

5. Conner M, Norman P. Predicting health behaviour: research and practice with social cognition models. Open University Press; 2005.

6. Müssener U, Bendtsen M, Karlsson N, White IR, McCambridge J, Bendtsen P. Effectiveness of Short Message Service Text-Based Smoking Cessation Intervention Among University Students. JAMA Internal Medicine. 2016;176(3):321.

7. Müssener U, Bendtsen M, Karlsson N, White IR, McCambridge J, Bendtsen P. SMS-based smoking cessation intervention among university students: study protocol for a randomised controlled trial (NEXit trial). Trials. 2015;16(1):140.

8. Thomas K, Bendtsen M, Linderoth C, Karlsson N, Bendtsen P, Müssener U. Short message service (SMS)-based intervention targeting alcohol consumption among university students: study protocol of a randomized controlled trial. Trials. 2017;18(1):156.

9. Thomas K, Müssener U, Linderoth C, Karlsson N, Bendtsen P, Bendtsen M. Effectiveness of a Text Messaging–Based Intervention Targeting Alcohol Consumption Among University Students: Randomized Controlled Trial. JMIR mHealth and uHealth. 2018;6(6):e146.

10. Müssener U, Bendtsen M, McCambridge J, Bendtsen P. User satisfaction with the structure and content of the NEXit intervention, a text messaging-based smoking cessation programme. BMC Public Health. 2016;16(1):1179.

11. Mussener U, Thomas K, Linderoth C, Leijon M, Bendtsen M. A Text Message-Based Intervention Targeting Alcohol Consumption Among University Students: User Satisfaction and Acceptability Study. JMIR human factors. 2018;5(3):e23.

12. Thomas K, Linderoth C, Bendtsen M, Bendtsen P, Mussener U. Text Message-Based Intervention Targeting Alcohol Consumption Among University Students: Findings From a Formative Development Study. JMIR mHealth and uHealth. 2016;4(4):e119.

13. Michie S, Richardson M, Johnston M, Abraham C, Francis J, Hardeman W, et al. The Behavior Change Technique Taxonomy (v1) of 93 Hierarchically Clustered Techniques: Building an International Consensus for the Reporting of Behavior Change Interventions. Annals of Behavioral Medicine. 2013 Aug;46(1):81–95.

14. Michie S, Whittington C, Hamoudi Z, Zarnani F, Tober G, West R. Identification of behaviour change techniques to reduce excessive alcohol consumption. Addiction (Abingdon, England). 2012 Aug;107(8):1431–40.

15. Bendtsen P, McCambridge J, Bendtsen M, Karlsson N, Nilsen P. Effectiveness of a Proactive Mail-Based Alcohol Internet Intervention for University Students: Dismantling the Assessment and Feedback Components in a Randomized Controlled Trial. Journal of Medical Internet Research. 2012 Oct 31;14(5):e142.

16. McCambridge J, Bendtsen M, Karlsson N, White IR, Nilsen P, Bendtsen P. Alcohol assessment and feedback by email for university students: Main findings from a randomised controlled trial. British Journal of Psychiatry. 2013;203(5):334–40.

17. Bendtsen P, Bendtsen M, Karlsson N, White IR, McCambridge J. Online Alcohol Assessment and Feedback for Hazardous and Harmful Drinkers: Findings From the AMADEUS-2 Randomized Controlled Trial of Routine Practice in Swedish Universities. Journal of Medical Internet Research. 2015;17(7):e170.

For peer review only

18. Michie S, Abraham C, Whittington C, Mcateer J. Effective Techniques in Healthy Eating and Physical Activity Interventions : A Meta-Regression. 2009;28(6):690–701.

19. Murray JM, Brennan SF, French DP, Patterson CC, Kee F, Hunter RF. Effectiveness of physical activity interventions in achieving behaviour change maintenance in young and middle aged adults: A systematic review and meta-analysis. Social science & medicine (1982). 2017 Nov;192:125–33.

20. Knittle K, Nurmi J, Crutzen R, Hankonen N, Beattie M, Dombrowski SU. How can interventions increase motivation for physical activity? A systematic review and meta-analysis. Health psychology review. 2018 Sep;12(3):211–30.

21. Howlett N, Trivedi D, Troop NA, Chater AM. Are physical activity interventions for healthy inactive adults effective in promoting behavior change and maintenance, and which behavior change techniques are effective? A systematic review and meta-analysis. Translational behavioral medicine. 2019 Jan;9(1):147–57.

22. Ashton LM, Sharkey T, Whatnall MC, Williams RL, Bezzina A, Aguiar EJ, et al. Effectiveness of Interventions and Behaviour Change Techniques for Improving Dietary Intake in Young Adults: A Systematic Review and Meta-Analysis of RCTs. Nutrients. 2019 Apr;11(4).

23. Garnett C V, Crane D, Brown J, Kaner EFS, Beyer FR, Muirhead CR, et al. Behavior Change Techniques Used in Digital Behavior Change Interventions to Reduce Excessive Alcohol Consumption: A Meta-regression. Annals of behavioral medicine : a publication of the Society of Behavioral Medicine. 2018 May;52(6):530–43.

24. McCrabb S, Baker AL, Attia J, Skelton E, Twyman L, Palazzi K, et al. Internet-Based Programs Incorporating Behavior Change Techniques Are Associated With Increased Smoking Cessation in the General Population: A Systematic Review and Meta-analysis. Annals of behavioral medicine : a publication of the Society of Behavioral Medicine. 2019 Feb;53(2):180–95.

25. Ek A, Alexandrou C, Söderström E, Bergman P, Delisle Nyström C, Direito A, et al. Effectiveness of a 3-month mobile phone based behavior change program on active transportation and physical activity in adults: A randomized controlled trial. JMIR mHealth uHealth. 2020;8(6).

26. Newby K, Teah G, Cooke R, Li X, Brown K, Salisbury-Finch B, et al. Do automated digital health behaviour change interventions have a positive effect on self-efficacy? A systematic review and meta-analysis. Health Psychology Review. 2020 Jan 20;1–19.

27. Crane RS, Brewer J, Feldman C, Kabat-Zinn J, Santorelli S, Williams JMG, et al. What defines mindfulness-based programs? The warp and the weft. Psychological Medicine. 2017 Apr 29;47(6):990–9.

28. Creswell JD. Mindfulness Interventions. Annual Review of Psychology. 2017 Jan 3;68(1):491–516.

29. Wong SYS, Chan JYC, Zhang D, Lee EKP, Tsoi KKF. The Safety of Mindfulness-Based Interventions: a Systematic Review of Randomized Controlled Trials. Mindfulness. 2018 Oct 2;9(5):1344–57.

30. Galante J, Friedrich C, Dawson AF, Modrego-Alarcón M, Gebbing P, Delgado-Suárez I, et al. Mindfulness-based programmes for mental health promotion in adults in nonclinical settings: A systematic review and meta-analysis of randomised controlled trials. Patel V, editor. PLOS Medicine. 2021 Jan 11;18(1):e1003481.

31. Neff KD, Germer CK. A Pilot Study and Randomized Controlled Trial of the Mindful Self-Compassion Program. Journal of Clinical Psychology. 2013 Jan;69(1):28–44.

32. Bendtsen M, McCambridge J. Reducing Alcohol Consumption Among Risky Drinkers in the General Population of Sweden Using an Interactive Mobile Health Intervention: Protocol for a Randomized Controlled Trial. JMIR Research Protocols. 2019;8(4):e13119.

For peer review only

For peer review only

STANDARD PROTOCOL ITEMS: RECOMMENDATIONS FOR INTERVENTIONAL TRIALS

SPIRIT 2013 Checklist: Recommended items to address in a clinical trial protocol and related documents*

Section/item	Item No	Description	Addressed on page number
**Administrative information**			
Title	1	Descriptive title identifying the study design, population, interventions, and, if applicable, trial acronym	______1______
Trial registration	2a	Trial identifier and registry name. If not yet registered, name of intended registry	_____1,2______
	2b	All items from the World Health Organization Trial Registration Data Set	_____1,2______
Protocol version	3	Date and version identifier	______NA____
Funding	4	Sources and types of financial, material, and other support	_____ 14_____
Roles and responsibilities	5a	Names, affiliations, and roles of protocol contributors	_____ 1______
	5b	Name and contact information for the trial sponsor	_____14____
	5c	Role of study sponsor and funders, if any, in study design; collection, management, analysis, and interpretation of data; writing of the report; and the decision to submit the report for publication, including whether they will have ultimate authority over any of these activities	_____14____
	5d	Composition, roles, and responsibilities of the coordinating centre, steering committee, endpoint adjudication committee, data management team, and other individuals or groups overseeing the trial, if applicable (see Item 21a for data monitoring committee)	_____NA_____

For peer review only

**Introduction**			
Background and rationale	6a	Description of research question and justification for undertaking the trial, including summary of relevant studies (published and unpublished) examining benefits and harms for each intervention	_____3______
	6b	Explanation for choice of comparators	_____3_______
Objectives	7	Specific objectives or hypotheses	_____3_______
Trial design	8	Description of trial design including type of trial (eg, parallel group, crossover, factorial, single group), allocation ratio, and framework (eg, superiority, equivalence, noninferiority, exploratory)	_____4______
**Methods: Participants, interventions, and outcomes**			
Study setting	9	Description of study settings (eg, community clinic, academic hospital) and list of countries where data will be collected. Reference to where list of study sites can be obtained	_____4_______
Eligibility criteria	10	Inclusion and exclusion criteria for participants. If applicable, eligibility criteria for study centres and individuals who will perform the interventions (eg, surgeons, psychotherapists)	_____4______
Interventions	11a	Interventions for each group with sufficient detail to allow replication, including how and when they will be administered	____4,5_______
	11b	Criteria for discontinuing or modifying allocated interventions for a given trial participant (eg, drug dose change in response to harms, participant request, or improving/worsening disease)	_____NA______
	11c	Strategies to improve adherence to intervention protocols, and any procedures for monitoring adherence (eg, drug tablet return, laboratory tests)	_____NA_____
	11d	Relevant concomitant care and interventions that are permitted or prohibited during the trial	_____NA______
Outcomes	12	Primary, secondary, and other outcomes, including the specific measurement variable (eg, systolic blood pressure), analysis metric (eg, change from baseline, final value, time to event), method of aggregation (eg, median, proportion), and time point for each outcome. Explanation of the clinical relevance of chosen efficacy and harm outcomes is strongly recommended	_____5,6______
Participant timeline	13	Time schedule of enrolment, interventions (including any run-ins and washouts), assessments, and visits for participants. A schematic diagram is highly recommended (see Figure)	__6, Figure 1__

For peer review only - http://bmjopen.bmj.com/site/about/guidelines.xhtml

Sample size	14	Estimated number of participants needed to achieve study objectives and how it was determined, including clinical and statistical assumptions supporting any sample size calculations	_____8.9______
Recruitment	15	Strategies for achieving adequate participant enrolment to reach target sample size	_____8.9______

Methods: Assignment of interventions (for controlled trials)

Allocation:

Sequence generation	16a	Method of generating the allocation sequence (eg, computer-generated random numbers), and list of any factors for stratification. To reduce predictability of a random sequence, details of any planned restriction (eg, blocking) should be provided in a separate document that is unavailable to those who enrol participants or assign interventions	______7_______
Allocation concealment mechanism	16b	Mechanism of implementing the allocation sequence (eg, central telephone; sequentially numbered, opaque, sealed envelopes), describing any steps to conceal the sequence until interventions are assigned	_____7________
Implementation	16c	Who will generate the allocation sequence, who will enrol participants, and who will assign participants to interventions	_____7________
Blinding (masking)	17a	Who will be blinded after assignment to interventions (eg, trial participants, care providers, outcome assessors, data analysts), and how	_____7________
	17b	If blinded, circumstances under which unblinding is permissible, and procedure for revealing a participant's allocated intervention during the trial	_____7________

Methods: Data collection, management, and analysis

Data collection methods	18a	Plans for assessment and collection of outcome, baseline, and other trial data, including any related processes to promote data quality (eg, duplicate measurements, training of assessors) and a description of study instruments (eg, questionnaires, laboratory tests) along with their reliability and validity, if known. Reference to where data collection forms can be found, if not in the protocol	____6_________
	18b	Plans to promote participant retention and complete follow-up, including list of any outcome data to be collected for participants who discontinue or deviate from intervention protocols	_____6________

Data management	19	Plans for data entry, coding, security, and storage, including any related processes to promote data quality (eg, double data entry; range checks for data values). Reference to where details of data management procedures can be found, if not in the protocol	___NA_________
Statistical methods	20a	Statistical methods for analysing primary and secondary outcomes. Reference to where other details of the statistical analysis plan can be found, if not in the protocol	____7,8________
	20b	Methods for any additional analyses (eg, subgroup and adjusted analyses)	____7,8________
	20c	Definition of analysis population relating to protocol non-adherence (eg, as randomised analysis), and any statistical methods to handle missing data (eg, multiple imputation)	____7,8_______
**Methods: Monitoring**			
Data monitoring	21a	Composition of data monitoring committee (DMC); summary of its role and reporting structure; statement of whether it is independent from the sponsor and competing interests; and reference to where further details about its charter can be found, if not in the protocol. Alternatively, an explanation of why a DMC is not needed	_____NA______
	21b	Description of any interim analyses and stopping guidelines, including who will have access to these interim results and make the final decision to terminate the trial	_____8.9______
Harms	22	Plans for collecting, assessing, reporting, and managing solicited and spontaneously reported adverse events and other unintended effects of trial interventions or trial conduct	_____8,9_______
Auditing	23	Frequency and procedures for auditing trial conduct, if any, and whether the process will be independent from investigators and the sponsor	_____NA_______
**Ethics and dissemination**			
Research ethics approval	24	Plans for seeking research ethics committee/institutional review board (REC/IRB) approval	______10_____
Protocol amendments	25	Plans for communicating important protocol modifications (eg, changes to eligibility criteria, outcomes, analyses) to relevant parties (eg, investigators, REC/IRBs, trial participants, trial registries, journals, regulators)	______NA_____

Consent or assent	26a	Who will obtain informed consent or assent from potential trial participants or authorised surrogates, and how (see Item 32)	_____4_______
	26b	Additional consent provisions for collection and use of participant data and biological specimens in ancillary studies, if applicable	_____4______
Confidentiality	27	How personal information about potential and enrolled participants will be collected, shared, and maintained in order to protect confidentiality before, during, and after the trial	_____NA______
Declaration of interests	28	Financial and other competing interests for principal investigators for the overall trial and each study site	_____14_____
Access to data	29	Statement of who will have access to the final trial dataset, and disclosure of contractual agreements that limit such access for investigators	_____14______
Ancillary and post-trial care	30	Provisions, if any, for ancillary and post-trial care, and for compensation to those who suffer harm from trial participation	_____NA______
Dissemination policy	31a	Plans for investigators and sponsor to communicate trial results to participants, healthcare professionals, the public, and other relevant groups (eg, via publication, reporting in results databases, or other data sharing arrangements), including any publication restrictions	_____10______
	31b	Authorship eligibility guidelines and any intended use of professional writers	_____14______
	31c	Plans, if any, for granting public access to the full protocol, participant-level dataset, and statistical code	_____14_____
**Appendices**			
Informed consent materials	32	Model consent form and other related documentation given to participants and authorised surrogates	_____NA___
Biological specimens	33	Plans for collection, laboratory evaluation, and storage of biological specimens for genetic or molecular analysis in the current trial and for future use in ancillary studies, if applicable	_____NA______

*It is strongly recommended that this checklist be read in conjunction with the SPIRIT 2013 Explanation & Elaboration for important clarification on the items. Amendments to the protocol should be tracked and dated. The SPIRIT checklist is copyrighted by the SPIRIT Group under the Creative Commons "Attribution-NonCommercial-NoDerivs 3.0 Unported" license.

BMJ Open

Digital multiple health behaviour change intervention targeting online help seekers: protocol for the Coach randomised factorial trial

Journal:	*BMJ Open*
Manuscript ID	bmjopen-2022-061024.R1
Article Type:	Protocol
Date Submitted by the Author:	13-May-2022
Complete List of Authors:	Åsberg, Katarina; Linköping University, Department of Health, Medicine and Caring Sciences<br>Blomqvist, Jenny; Linköping University, Department of Health, Medicine and Caring Sciences<br>Lundgren, Oskar; Linköping University, Department of Health, Medicine and Caring Sciences<br>Henriksson, Hanna; Linköping University, Department of Health, Medicine and Caring Sciences<br>Henriksson, Pontus; Linköping University, Department of Health, Medicine and Caring Sciences<br>Bendtsen, Preben; Linköping University, Department of Health, Medicine and Caring Sciences; Motala Hospital, Department of Medical Specialist<br>Löf, Marie; Linköping University, Department of Health, Medicine and Caring Sciences; Karolinska Institutet, Department of Biosciences and Nutrition<br>Bendtsen, Marcus; Linköping University, Department of Health, Medicine and Caring Sciences
**Primary Subject Heading**:	Public health
Secondary Subject Heading:	Epidemiology
Keywords:	PUBLIC HEALTH, PREVENTIVE MEDICINE, EPIDEMIOLOGY

SCHOLARONE™
Manuscripts

DIGITAL MULTIPLE HEALTH BEHAVIOUR CHANGE INTERVENTION TARGETING ONLINE HELP SEEKERS: PROTOCOL FOR THE *COACH* RANDOMISED FACTORIAL TRIAL

Katarina Åsberg[1], Jenny Blomqvist[1], Oskar Lundgren[1], Hanna Henriksson[1], Pontus Henriksson[1], Preben Bendtsen[1,2], Marie Löf[1,3], Marcus Bendtsen[1,*]

[1] Department of Health, Medicine and Caring Sciences, Linköping University, Sweden.
[2] Department of Medical Specialist, Motala, Sweden.
[3] Department of Biosciences and Nutrition, Karolinska Institutet, Sweden.

*Corresponding author
Marcus Bendtsen (marcus.bendtsen@liu.se)
Department of Health, Medicine and Caring Sciences, Division of Society and Health
Linköping University, 581 83 Linköping, registrator@liu.se, +46 13 28 10 00

Trial registration: Prospectively registered in ISRCTN (ISRCTN16420548)
https://www.isrctn.com/ISRCTN16420548

Ethical approval: The study was approved by the Swedish Ethical Review Authority on 2021-08-11 (Dnr 2021-02855).

Word count: 4466

For peer review only

ABSTRACT

Introduction: Unhealthy lifestyle behaviours continue to be highly prevalent, including alcohol consumption, unhealthy diets, insufficient physical activity, and smoking. There is a lack of effective interventions which have a large enough reach into the community to improve public health. Additionally, the common co-occurrence of multiple unhealthy behaviours demands investigation of efforts which address more than single behaviours.

Methods and analysis: The effects of six components of a novel digital multiple health behaviour change intervention on alcohol consumption, diet, physical activity, and smoking (co-primary outcomes), will be estimated in a factorial randomised trial. The components are designed to facilitate behaviour change, e.g., through goal setting or increasing motivation, and are either present or absent depending on allocation (i.e., 6 factors with 2 levels each). The study population will be those seeking help online, recruited through search engines, social media, and lifestyle related websites. Included will be those who are at least 18 years of age and have at least one unhealthy behaviour. An adaptive design will be used to periodically make decisions to continue or stop recruitment, with simulations suggesting a final sample size between 1500 and 2500 participants. Multilevel regression models will be used to analyse behavioural outcomes collected at 2- and 4-months post-randomisation.

Ethics and dissemination: Approved by the Swedish Ethical Review Authority on 2021-08-11 (Dnr 2021-02855). Since participation is likely motivated by gaining access to novel support, the main concern is de-motivation and opportunity cost if the intervention is found to only exert small effects. Recruitment began on 2021-10-19, with an anticipated recruitment period of 12 months.

Registration: Prospectively registered on 2021-10-05 (ISRCTN: ISRCTN16420548). The methods of this study were pre-registered prior to enrollment commenced on the Open Science Platform on 2021-10-19 (https://osf.io/xyj3p/).

ARTICLE SUMMARY

STRENGTHS AND LIMITATIONS OF THIS STUDY

- Pragmatic recruitment of individuals seeking help online to a factorial trial allow for dismantling of the effectiveness of the components which make up a digital multiple health behaviour change intervention.
- An adaptive trial design reduces the risk of under- and over-recruitment of participants.
- Despite double blind procedures, research participation effects may affect self-reported outcomes and introduce bias.
- Single face-valid items used to measure mediators reduce participant burden but may limit the interpretation of findings.

For peer review only - http://bmjopen.bmj.com/site/about/guidelines.xhtml

INTRODUCTION

Behavioural risk factors, such as harmful alcohol consumption, unhealthy diets, insufficient physical activity, and smoking, contribute to about a third of global disability adjusted life years, and are leading causes of non-communicable diseases (NCDs), including cardiovascular disease, respiratory disease, cancer, and diabetes (1,2). The World Health Organization has determined that reducing the prevalence of behavioural risk factors should be a priority in many societies to reduce the incidence of NCDs and disability adjusted life years (3). It is therefore important that effective and scalable means of helping individuals to improve their health behaviours are established.

The Public Health Agency of Sweden's national public health survey from 2020 (4) (n = 16 947) reports data on lifestyle behaviours of Swedish citizens aged 16-84. According to the survey, 16% of respondents report hazardous or harmful alcohol consumption, 35% report being insufficiently physically active, 12% report smoking occasionally or daily, and 93% report eating less fruit and vegetables than recommended. Additionally, 52% of individuals report being obese or overweight. Unfortunately, with the exception of smoking, the prevalence rates of these behaviours have not decreased markedly over the past 10 years, with some increasing, witnessing of a lost decade for prevention efforts.

For prevention efforts to have an impact on the general population, they need to have extensive reach among those who may benefit. No single setting will be able to achieve this, e.g., only 1-5% of individuals visiting primary health care clinics in Sweden are given advice with respect to their lifestyle (5), despite many more in need of such advice. Unhealthy lifestyle behaviours also tend to cluster and interact (6,7), e.g. those who are overweight are more likely to be physically inactive, and excessive alcohol consumption may lead to weight gain. Risks from multiple unhealthy lifestyle behaviours may be multiplicative (8); thus, it is of value to not only extend the reach of interventions, but to also investigate tools designed to support change of multiple health behaviours.

One way of reaching further into the community with a multiple health behaviour change intervention is to offer digital support tools to those searching online for help. This is especially promising in Sweden, since the internet is used daily by approximately 90% of the population, and the same proportion use smartphones on a regular basis (9,10). A recent effectiveness trial of a digital alcohol intervention among online help-seekers in Sweden found evidence of positive effects on alcohol consumption (11), but also that only 13.5% of study participants turned off the support, which indicates that receiving support for behaviour change through digital means is an acceptable method for many. Studies evaluating digital interventions addressing *multiple* health behaviours have also shown promising results (12–15). However, the evidence of these types of interventions in more general populations is lacking, as the majority of studies have been conducted among university students, employees within specific fields, or patients with specific health conditions. In addition, behaviour interventions often consist of several components or modules, yet are commonly evaluated as a whole (16), leaving a paucity of evidence for the effects of the dismantled components. Increasing our understanding of the effects at the component level, in particular with respect to multiple behaviours, may help move the field of behaviour interventions forward.

OBJECTIVES

This study aims to estimate the effects of the components of a digital intervention on multiple health behaviours (alcohol, physical activity, diet, and smoking) among individuals seeking help online. The objectives of the study are to:

1. Estimate the effects of a digital intervention's different components on individual health behaviours:
    a. Weekly alcohol consumption and number of episodes per month of heavy drinking.

For peer review only - http://bmjopen.bmj.com/site/about/guidelines.xhtml

   b. Average daily fruit and vegetable consumption.

   c. Weekly moderate to vigorous physical activity.

   d. Four-week point prevalence of smoking.

2. Estimate the degree to which the effects of the components are mediated through perceived importance, confidence, and know-how.

3. Detect interactions among health behaviour change, e.g., those who stop smoking may also reduce their alcohol consumption, and the degree to which this is moderated by the components of the intervention.

METHODS

A double blind factorial randomised trial (17) (6 factors with 2 levels each) will be employed to address the objectives of the study. A Bayesian group sequential design will be employed to periodically make decisions to continue or stop recruitment (18–20). This protocol contains relevant items from the *Standard Protocol Items: Recommendations for Interventional Trials* (SPIRIT) (21). The methods of this trial, including the statistical analysis plan, was pre-registered on the Open Science Platform prior to enrollment commenced (https://osf.io/xyj3p/).

STUDY SETTING, RECRUITMENT AND ELIGIBILITY

We will recruit individuals seeking information about health and behaviour change by advertising on Google, Bing, and Facebook (restricted to Sweden), as well as on websites which focus on lifestyle and behaviour change (e.g., livsstilsanalys.se). Individuals exposed to the advert will be advised to sign up to the study by sending a text message with a specific code to a dedicated phone number.

Within 10 minutes, individuals will receive a text message with a hyperlink that takes them to a web page with informed consent materials. Consent will be given by clicking on a button on the bottom of the page. All individuals giving informed consent will be asked to complete a baseline questionnaire, which will also assess eligibility for the trial (please see Appendix A). Individuals will be included in the trial if they fulfil at least one of five conditions:

- **Weekly alcohol consumption:** Consumed 10/15 (female/male) or more standard drinks of alcohol the past week. A standard drink of alcohol is in Sweden defined as 12 grams of pure alcohol.

- **Heavy episodic drinking:** Consumed 4/5 (female/male) or more standard drinks of alcohol on a single occasion at least once the past month.

- **Fruit and vegetables:** Consumed less than 500 grams of fruit and vegetables on average per day the past week.

- **Moderate to vigorous physical activity:** Spent less than 150 minutes on moderate to vigorous physical activity the past week.

- **Smoking:** Having smoked at least one cigarette the past week.

Individuals will be explicitly excluded if they do not fulfil any of the criteria or if they are less than 18 years of age. The trial information and intervention will be entirely in Swedish and delivered to participants' mobile phones, thus not comprehending Swedish well enough to sign up or not having access to a mobile phone will implicitly exclude individuals.

For peer review only - http://bmjopen.bmj.com/site/about/guidelines.xhtml

INTERVENTIONS

The digital intervention, which is called *Coach*, consists of six components which users access using their mobile phone, based on an intervention design we have used previously (22,23). The intervention is designed around social cognitive theories of behaviour change, with a focus on modifying environment, intention, and skills (24,25). The intervention's components are intended to be used as a toolbox, allowing users to choose which parts of the intervention to interact with and tailor the support to their needs. Participants eligible for the trial will be allocated to one of 64 factorial conditions, each condition representing a unique combination of the six components - which are either present or absent (2^6 = 64 conditions). The intervention materials can be accessed at participants' discretion over a 4-month period, and each Sunday afternoon participants will receive a text message with a link and a reminder to access the intervention materials. A summary of the components is presented in Table 1, and a detailed description of the six components is available in Appendix B.

Table 1 - Brief description of the six components of the Coach intervention

**Screening and feedback**	**Present / Absent**
Every Sunday afternoon, participants will receive a text message with a hyperlink which takes them to a questionnaire regarding their current health behaviours. Once complete, feedback on their current behaviour is given in relation to national guidelines. Thereafter users are given access to the rest of the components (depending on allocation).	When absent participants will not be shown the questionnaire but instead only national guidelines without personal feedback.
**Goalsetting and planning**	
This component let participants set a goal for their future behaviour and plan for what to do when they struggle and succeed. Participants can also accept challenges for the coming week, e.g., to walk for 15 minutes each day, or to not drink any alcohol this week. Self-composed challenges are also available. Reminders are sent via texts to participants about their goals and challenges throughout the week.	When absent, this component will not be visible.
**Motivation**	
This component contains information and tools to increase participants' motivation for change. This includes information on negative health consequences, costs induced from certain behaviours, and reflective tasks. If participants choose, they can also activate motivational text messages which are sent to them throughout the week.	When absent, this component will not be visible, and text messages will not be available.
**Skills and know-how**	
Concrete tips on how to initiate and maintain change in everyday life is offered in this component. This includes giving participants strategies they can use to say no to alcoholic beverages at parties, how to increase the nutritional value of their breakfast, etc. If participants choose, they can also activate text messages with tips sent to them throughout the week.	When absent, this component will not be visible, and text messages will not be available.
**Mindfulness**	
This component aims to increase users' awareness of their own lived experience and strengthen their capacity for non-reactive, compassionate, and less stressful way of being in the world. Mindfulness exercises are offered to participants, including guided meditations.	When absent, this component will not be visible, and guided meditations not available.
**Self-composed text messages**	
Participants are given the opportunity to compose messages and have them sent to themselves throughout the week (on days and times of their own choosing). A participant may for instance write a message to themselves reminding them to eat two fruits each day, to not drink anything on Wednesdays, or to go for a walk with a friend.	When absent, this component will not be visible.

For peer review only - http://bmjopen.bmj.com/site/about/guidelines.xhtml

OUTCOMES

MEASURES

Outcomes are listed here and subsequently explained. All questionnaires (baseline, 1-, 2- and 4-month follow-up) used in the trial can be found in Appendix A.

Primary outcome measures

- **Alcohol:** Weekly alcohol consumption; monthly frequency of heavy episodic drinking.
- **Diet:** Average daily consumption of fruit and vegetables.
- **Physical activity:** Weekly moderate to vigorous physical activity (MVPA).
- **Smoking:** Four-week point prevalence of smoking abstinence.

Secondary outcome measures

- Perceived stress.
- Weekly consumption of sugary drinks.
- Weekly consumption of candy and snacks.
- Body mass index (BMI).
- Weekly number of cigarettes smoked.
- Quality of life (QoL).

Mediation measures

- Importance of change.
- Confidence in one's ability to change.
- Knowledge of how to change.

For peer review only

PRIMARY AND SECONDARY OUTCOMES

Weekly alcohol consumption will be assessed by asking participants the number of standard drinks of alcohol they consumed last week (short term recall method (26)). Frequency of heavy episodic drinking will be assessed by asking participants how many times they have consumed 4/5 (female/male) or more standard drinks of alcohol on one occasion the past month. These two outcomes are both part of the proposed core outcome set for brief alcohol interventions (27–29), and represent different risk behaviours which are sometimes found in the same individual and sometimes not. For instance, one may have a high weekly alcohol consumption, and thereby be at risk for negative health consequences, without consuming 4/5 or more drinks on the same occasion. Similarly, having one episode of heavy episodic drinking increases the risk of short-term

For peer review only - http://bmjopen.bmj.com/site/about/guidelines.xhtml

consequences (such as injury) and long term health consequences, but does not fulfil the criteria for total weekly consumption.

Diet and physical activity will be measured utilising a questionnaire based on the previously published questionnaire by the National Board of Health and Welfare in Sweden (7), and was further modified to also include portion sizes. The consumption of fruit and vegetables will be measured using two questions concerning the number of portions (100 g) of fruit and vegetables (respectively) the participants ate on average per day during the past week. Sugary drinks consumption will be measured by a question regarding the number of units (33 cl) of sugary drinks participants consumed the past week, and candy and snacks will be measured using a single question regarding number of servings consumed last week. MVPA will be estimated by summing responses to two questions regarding the number of minutes spent on moderate and vigorous physical activity, respectively, during the past week.

Body mass index will be measured by asking participants to report their weight and height.

Four-week point prevalence of smoking abstinence (no cigarettes the past four weeks) will be asked as a binary question. This is a suggested measure by the Society of Research on Nicotine and Tobacco (30). Participants who have smoked any cigarette the past four weeks will be asked for the number of cigarettes smoked the past week.

QoL will be measured using PROMIS Global 10 (31), both to estimate the degree to which intervention components effect QoL but also for health economic evaluations. Perceived stress will be assessed using the short form perceived stress scale (PSS-4) (32).

MEDIATION MEASURES

Participants will be asked to report on confidence, importance, and know-how; which are three psychosocial factors believed to be important markers of behaviour change (24,25,33–35). To reduce participant burden, we will use single face-valid items, acknowledging the limitation of such measures.

PARTICIPANT TIMELINE AND FOLLOW-UPS

A trial participant timeline is presented in Figure 1. Intervention components (depending on allocation) will be made available to participants all at once and stay available to participants at their own discretion throughout the 4-month period (with weekly reminders). There are 3 follow-up stages: 1-, 2-, and 4-months post randomisation. All follow-ups will be initiated by sending text messages to participants with hyperlinks to questionnaires. The following additional attempts will be made to collect data:

1. A total of two text reminders will be sent two days apart to those who have not responded.
2. If there is no response to the mediator questions at the 1-month follow-up, then the questions will be sent in a text message and participants are asked to respond directly with a text.
3. If there is no response to the 2- and 4-month follow-ups, then we will call participants to collect responses for the primary outcome measures only. A maximum of 5 call attempts will be made.

INSERT FIGURE 1 HERE

Figure 1 - SPIRIT figure showing participant timeline throughout the study

For peer review only

ASSIGNMENT OF INTERVENTIONS

Randomisation will be fully automated and computerised. Block randomisation will be used to allocate participants to the 64 conditions (random block sizes of 64 and 128). Neither research personnel nor participants will be able to influence allocation.

Research personnel will be blind to allocation throughout the trial. All participants will have access to the intervention, albeit with different components, and they will not be made aware of the other available conditions and will therefore be blind to allocation.

PATIENT AND PARTICIPANT INVOLVEMENT STATEMENT

Outcome measures used in the trial are informed by national guidelines in Sweden, as well as those set by the WHO. Also, the Swedish National Board of Health and Welfare (7) have reported that research regarding multiple health behaviour change interventions is lacking. No patients or participants were involved in the planning of this trial or design of the intervention; however, both have been informed by our previous research involving individuals looking for help to change health related behaviours.

ANALYSIS

All analyses will be done keeping all participants in the groups to which they were randomised. Analyses will be done using both available data and imputation. Imputation will be done using multiple imputation with chained equations (36). The implicit missing at random (MAR) assumption underlying this approach will be investigated by two attrition analyses: (1) if data is missing systematically then it may be the case that early responders (answering without reminders) differ from non-responders (requiring several attempts), and in extension that late responders are more alike non-responders. Therefore, one attrition analysis will regress primary outcomes against number of attempts to collect follow-up before a response was recorded; (2) we will further explore the MAR assumption by investigating if responders and non-responders are different with respect to baseline characteristics.

Groups will be contrasted using multilevel regression models with covariates for group by component interactions and participant level adaptive intercepts. Models of longitudinal data (primary outcomes and perceived stress) will include group by time by component interactions. We will explore pairwise interactions among components. Bayesian inference will be used to estimate the parameters of the models (37–39) (see Sample Size for priors). For each coefficient of interest, we will report the marginal posterior probability of effect, and the median will be used as a point estimate of the magnitude of the effect. We will also report on 50% and 95% compatibility intervals.

MODELS

PRIMARY AND SECONDARY OUTCOMES

Analyses of primary outcomes will be conducted among those fulfilling the respective criteria for inclusion at baseline, e.g., weekly alcohol consumption will be analysed among those who reported having consumed 10/15 (female/male) or more units of alcohol the past week. BMI, sugary drinks, candy/snacks, QoL, and perceived stress will be analysed among all participants, and number of cigarettes smoked weekly among baseline smokers.

Weekly alcohol consumption, frequency of heavy episodic drinking per month, weekly intake of candy and snacks, number of sugary drinks per week, and cigarettes smoked per week are all count variables that are

For peer review only - http://bmjopen.bmj.com/site/about/guidelines.xhtml

likely skewed and over dispersed. Therefore, these outcomes will be analysed using negative binomial regression. If found not to be over dispersed, we will consider using normal regression (possibly log transformed). Average intake of fruit and vegetables per day, MVPA minutes per week, BMI, QoL, and perceived stress will be analysed using normal regression (possibly log transformed). Point prevalence of smoking abstinence will be analysed using logistic regression.

All models will be adjusted for age, sex, and mediators (importance, confidence, and know-how) at baseline. Primary outcomes and perceived stress will be adjusted for their respective baseline values, except for smoking prevalence which will be adjusted by the weekly number of cigarettes smoked at baseline. BMI, sugary drinks, and candy/snacks will be adjusted for baseline MVPA minutes per week and average intake of fruit and vegetables per day. Number of cigarettes smoked last week will be adjusted by its baseline value. QoL will be adjusted for perceived stress at baseline.

In addition to pairwise interactions between components, effect modification will be explored in all models to assess if any of the baseline characteristics moderate the effects of the components of the intervention.

MEDIATOR OUTCOMES

Mediators will be explored using a causal inference framework (40–42), using Bayesian inference to estimate the natural direct effect and natural indirect effect (as per the definitions of Pearl (42)). We will report on the posterior distributions of these two estimates, as well as the proportion of the total effect which is accounted for by the natural indirect effect. Four models will be created for each primary outcome measure, three which investigate the mediating factors on their own, and a fourth which incorporates all mediators at once. If any baseline characteristics were found to moderate the effects in the primary analysis, then additional mediator models will be created to include these as moderators.

INTERACTIONS AMONG HEALTH BEHAVIOURS

Outcome interactions, and determinants of such, will be investigated in an exploratory analysis. For instance, those who quit smoking may also be more likely to reduce their alcohol consumption, and this interaction may be moderated by baseline characteristics. In addition, we will investigate interactions between changes in perceived stress, QoL, and behaviour change. Models to detect such interactions will be explored and findings will be used to create hypotheses for future research.

SAMPLE SIZE

The trial will use a Bayesian group sequential design (18–20) to monitor recruitment with interim analyses planned for every 50 participants completing the 4-month follow-up. Each of the primary outcomes will be modelled according to the analysis plan (see Analysis), and coefficients for dummy variables representing presence/absence of each component at each follow-up interval will be assessed for effect, harm, and futility with respect to each outcome. We let $ß_{k,l,i}$ represent the regression coefficient for component *k*, at time *l*, for outcome *i*, and D all the data currently accumulated, then the target criteria will be:

- **Effect (fruit/veg. and physical activity):** $p(ß_{k,l,i} > 0 \mid D) > 97.5\%$ and $p(ß_{k,l,i} > 0.10 \mid D) > 50\%$
- **Harm (fruit/veg. and physical activity):** $p(ß_{k,l,i} < 0 \mid D) > 97.5\%$ and $p(ß_{k,l,i} < -0.10 \mid D) > 50\%$
- **Effect (alcohol and smoking):** $p(ß_{k,l,i} < 0 \mid D) > 97.5\%$ and $p(ß_{k,l,i} < -0.10 \mid D) > 50\%$
- **Harm (alcohol and smoking):** $p(ß_{k,l,i} > 0 \mid D) > 97.5\%$ and $p(ß_{k,l,i} > 0.10 \mid D) > 50\%$
- **Futility (all outcomes):** $p(-0.10 < ß_{k,l,i} < 0.10 \mid D) > 95\%$

For peer review only - http://bmjopen.bmj.com/site/about/guidelines.xhtml

Outcomes analysed using normal regression will be standardised when checking the above criteria. For the effect and harm criteria, we will use a standard normal prior for dummy covariates (mean = 0, sd = 1.0), and a slightly wider prior will be used for the futility criterion (mean = 0, sd = 2.0). The criteria should be viewed as targets, thus at each interim analysis we will evaluate each criterion and decide if we believe that recruitment should stop or continue. We will continue recruitment until one criterion is fulfilled for each component, for each outcome, at each follow-up interval. We will consider removing factors from the trial if the harm criteria are fulfilled for a component on all outcomes. We will not remove factors for which the effect or futility criteria are satisfied, as collecting additional data will facilitate reducing uncertainty regarding interaction effects. Note that we are estimating each component's effect on each outcome, thus we are not a-priori excluding any combination. If a component is ineffective with respect to a specific outcome, then this will be captured by the futility criteria, and will also be reported as a finding.

While the final sample size is not determined a-priori, we conducted a series of simulations with effect sizes at the minimal value of the above criteria (0.1 Cohen's d for fruit/veg and physical activity, 1.1 incidence rate ratios for alcohol, and 1.1 odds ratios for smoking). Simulations suggested that approximately 1500-2500 participants will be necessary to recruit. However, the criteria will decide, not the simulations. Despite having more conditions than in a traditional 2-arm trial (in this case 64 conditions), the factorial design is fully powered for each contrast (17). This can be understood by observing that half the study population are given access to each individual component (see Supplementary Appendix Table 1 in Appendix B), thus the other half creates a contrast (a type of control).

Note that the Bayesian approach allows us to make unlimited looks at the data without worrying about multiplicities and error rates, as would be necessary using a frequentist approach (43). Also, since no fixed effect size is pre-specified, we reduce the risk of stopping recruitment both too early and too late (20).

DISCUSSION

Maintaining a healthy diet and adequate physical exercise are proven ways to decrease the risk of many NCDs such as cancer and type II diabetes. More specifically, evidence suggests that the risk of many types of cancer is reduced by a diet which, among other things, includes vegetables and fruits and limits high-calorie foods and sugary drinks (44). Smoking has been identified as the most prominent risk factor for developing many types of cancer, however, there are indications that more complex connections are in effect. For instance, alcohol consumption is a strong risk factor for cancer in and of itself, however, it has a synergetic relation with smoking in the context of developing certain types of cancer, meaning that a combination of these health behaviours amounts to bigger risks than their individual effects (45,46). Research has provided strong evidence that risk factors for disease such as smoking, alcohol, physical inactivity and poor diet tend to have a clustered and co-occurring pattern in populations (47,48). Swedish data shows a similar tendency, increasing the risk of poor health outcomes in the population and hence providing additional incitement for future studies to utilise a multi-behaviour approach. Furthermore, previous research concludes the need for future research to use a holistic approach, focusing on multiple and simultaneous interventions for behavioural change (13,47,49–52)

Two meta-analyses reported modest effects of multiple health behaviour interventions in non-clinical (50) and clinical populations (53), with various suggested reasons, including poor implementation. Some of the limitations of past efforts may be difficult to overcome with traditional face-to-face interventions, due to the large demand on staff and other resources. Only 4 of the 69 trials in one of the meta-analyses (50) investigated the use of interventions delivered via digital technology (e.g., email, text messages or websites). These trials were however limited by low power or engagement, targeted university students or young individuals, and had questionable external validity. All in all, despite the extended reach which digital interventions may have, there is a lack of evidence for digital multiple health behaviour interventions targeting a more general population.

For peer review only - http://bmjopen.bmj.com/site/about/guidelines.xhtml

This factorial trial investigates the components of a novel multiple behaviour intervention. While our aim of the trial is to estimate the effects of the components on behaviour, we plan to conduct exploratory studies of engagement (54), which in combination with effect estimates will be used to determine future directions of study. Decisions to retain or remove components will therefore not be based solely on the statistical analyses in this study, but rather combined with engagement data and the evidence from the literature more widely. If for instance some components are found to exert only small effects, but was hardly used, we are more inclined to in future studies understand why it was not used and based on this redesign the component. On the other hand, components which are used often but still exert small effects may be candidates for replacement. If some components are found to only be effective for some behaviours, then these may be candidates for inclusion among those only with these unhealthy behaviours.

GENERALISABILITY AND LIMITATIONS

We have adopted a pragmatic recruitment strategy for this trial, using online channels, which closely mimics the way the intervention would be disseminated in a real-world context. The trial should therefore be viewed as estimating effectiveness of the intervention's components, rather than an efficacy. However, careful consideration should be taken due to the trial context creating expectations of and from participants (55,56), and those who take part in trials may be systematically different from those who do not. In addition, several limitations of the trial should be considered when interpreting findings.

The factorial design of this trial allows all participants to receive some support, even if some will receive a minimal number of components. Since conditions are unknown to participants we consider them blinded to allocation, which reduces the risk of bias (57,58). This does not however protect entirely against social desirability bias, as those who are positive to the treatment received may want to support its dissemination by reporting more positive outcomes than actual (59), which may be less likely if fewer components of the intervention are received. Compensatory rivalry bias could exacerbate this issue (60). We will ask questions with respect to participants' perceptions about the support received to support reasoning about the strength of these threats to validity.

Condition allocation may be revealed to research personnel when participants are called to collect follow-up data. This may be a source of bias, as non-blinded assessment of subjective measures have been found to bias estimates (61). Deducing the exact allocation is however unlikely, and personnel are instructed to not ask about anything else than the follow-up data. Using phone calls is a strategy employed to reduce the risk of attrition bias, which we believe outweighs the risk of detection bias.

Finally, there are two methodological compromises which are important to address. First, we use single face-valid items for mediators to reduce participant burden, which means that any marked mediation effect should be carefully interpreted to relate to the full concept of importance, confidence, and know-how. Second, criteria for stopping enrolment are based on the analysis of individual components which does not consider interactions among components. While it would be advantageous to include criteria for interactions, it is not practical to do so as it would increase the expected sample size markedly.

ETHICS AND DISSEMINATION

The study was approved by the Swedish Ethical Review Authority on 2021-08-11 (Dnr 2021-02855). Participants are likely to have been motivated to sign up for the trial by the potential of receiving novel support, leading to a risk of opportunity cost if the intervention only exerts small effects on behaviour. However, considering that current prevention efforts seem to not be enough to reduce the prevalence of unhealthy behaviours, and the potential effects and reach a digital multiple health behaviour change intervention could have among those seeking help online, this risk was deemed acceptable.

For peer review only

Recruitment began in October 2021, and we anticipate that recruitment will last no more than 12 months. A final dataset will therefore be available in January 2023, and findings will be subsequently submitted for peer-review in open access journals.

STATEMENTS

AUTHORS CONTRIBUTIONS

Study objectives and outcomes were decided by MB, ML, PB, PH, and HH. MB and KÅ designed the trial and analysis plan. Intervention materials were conceptualised and developed by KÅ, JB, MB, OL, ML, PB, PH, and HH, based on an intervention design by MB. MB, KÅ and JB drafted the protocol, which was revised by ML, PB, PH, HH and OL – all authors contributed with intellectual content and approved the final version. JB, KÅ, and MB will be responsible for data collection and statistical analysis. All authors will be responsible for communication of findings from the trial.

FUNDING

This trial has been funded by The Swedish Cancer Society (Cancerfonden, 20 0883 Pj, PI: Dr. Marcus Bendtsen), and is an extension of the MoBILE research program which is funded by the Swedish Research Council for Health, Working Life and Welfare (Grant number 2018-01410; PI: Prof. Marie Löf).

COMPETING INTERESTS

MB and PB own a private company (Alexit AB) that develops and distributes lifestyle behaviour interventions for use in healthcare settings. Alexit AB had no part in funding or planning of this trial but is relied upon for a service to send text messages.

DATA AVAILABILITY STATEMENT

Data will be made available to researchers upon reasonable request, after approval of a research proposal and signing of data transfer agreements.

REFERENCES

1. World health organization. Fact sheet Noncommunicable diseases. 2018.

2. Stanaway JD, Afshin A, Gakidou E, Lim SS, Abate D, Abate KH, et al. Global, regional, and national comparative risk assessment of 84 behavioural, environmental and occupational, and metabolic risks or clusters of risks for 195 countries and territories, 1990–2017: a systematic analysis for the Global Burden of Disease Stu. The Lancet. 2018 Nov;392(10159):1923–94.

3. World Health Organization. Global action plan for the prevention and control of noncommunicable diseases 2013-2020. 2013.

4. Folkhälsomyndigheten. Bilaga 1 till ”Folkhälsans utveckling – Årsrapport 2021” - Resultat i tabellform [Internet]. 2021. Available from: https://www.folkhalsomyndigheten.se/globalassets/publicerat-material/publikationer/folkhalsan-arsrapport-2021/folkhalsans-utveckling-arsrapport-2021-bilaga-1-21014-1.pdf

5. Socialstyrelsen. Primärvårdens arbete med prevention och behandling av ohälsosamma levnadsvanor 2016. 2017.

For peer review only

6. Schuit AJ, Van Loon AJM, Tijhuis M, Ocké MC. Clustering of lifestyle risk factors in a general adult population. Prev Med. 2002;35(3):219–24.

7. Socialstyrelsen. Nationella riktlinjer för prevention och behandling vid ohälsosamma levnadsvanor. 2018.

8. Myint PK, Luben RN, Wareham NJ, Bingham SA, Khaw K tee. Combined effect of health behaviours and risk of first ever Norfolk cohort of European Prospective Investigation of Cancer (EPIC Norfolk): prospective population study. 2009;338(1):b349.

9. Befolkningens it-användning 2020. SCB; 2020.

10. Svenskarna och internet. Internet Stiftelsen; 2021.

11. Bendtsen M, Åsberg K, McCambridge J. Effectiveness of a digital intervention versus alcohol information for online help-seekers in Sweden: a randomised controlled trial. BMC Medicine. 2022;In-press.

12. Deitz D, Cook RF, Hersch RK, Leaf S. Heart healthy online: an innovative approach to risk reduction in the workplace. J Occup Environ Med. 2014;56(5):547–53.

13. Schulz DN, Kremers SPJ, Vandelanotte C, Van Adrichem MJG, Schneider F, Candel MJJM, et al. Effects of a web-based tailored multiple-lifestyle intervention for adults: A two-year randomized controlled trial comparing sequential and simultaneous delivery modes. J Med Internet Res. 2014;16(1):e26.

14. Duncan MJ, Vandelanotte C, Trost SG, Rebar AL, Rogers N, Burton NW, et al. Balanced: a randomised trial examining the efficacy of two self-monitoring methods for an app-based multi-behaviour intervention to improve physical activity, sitting and sleep in adults. BMC Public Health. 2016 Dec;16(1):670.

15. A O, J B, A D, de Weerdt I, de Vries H, Oenema A, et al. Efficacy and use of an internet-delivered computer-tailored lifestyle intervention, targeting saturated fat intake, physical activity and smoking cessation: a randomized controlled trial. Ann Behav Med. 2008;35(2):125–35.

16. Pham Q, Wiljer D, Cafazzo JA. Beyond the Randomized Controlled Trial: A Review of Alternatives in mHealth Clinical Trial Methods. JMIR MHealth UHealth. 2016 Sep 9;4(3):e107.

17. Montgomery AA, Peters TJ, Little P. Design, analysis and presentation of factorial randomised controlled trials. BMC Med Res Methodol. 2003 Dec 24;3(1):26.

18. Gsponer T, Gerber F, Bornkamp B, Ohlssen D, Vandemeulebroecke M, Schmidli H. A practical guide to Bayesian group sequential designs. Pharm Stat. 2014;13(1):71–80.

19. Berry DA. Bayesian clinical trials. Nat Rev Drug Discov. 2006 Jan;5(1):27–36.

20. Bendtsen M. The P Value Line Dance: When Does the Music Stop? J Med Internet Res. 2020;22(8):e21345.

21. Chan AW, Tetzlaff JM, Altman DG, Laupacis A, Gøtzsche PC, Krleža-Jerić K, et al. SPIRIT 2013 Statement: Defining Standard Protocol Items for Clinical Trials. Ann Intern Med. 2013 Feb 5;158(3):200.

22. Bendtsen M, McCambridge J. Reducing Alcohol Consumption Among Risky Drinkers in the General Population of Sweden Using an Interactive Mobile Health Intervention: Protocol for a Randomized Controlled Trial. JMIR Res Protoc. 2019;8(4):e13119.

23. Åsberg K, Lundgren O, Henriksson H, Henriksson P, Bendtsen P, Löf M, et al. Multiple lifestyle behaviour mHealth intervention targeting Swedish college and university students: protocol for the *Buddy* randomised factorial trial. BMJ Open. 2021 Dec;11(12):e051044.

24. Fishbein M, Triandis HC, Kanfer FH, Becker M, Middlestadt SE, Eichler A. Factors influencing behaviour and behaviour change. In: Handbook of Health Psychology. Psychology Press Taylor & Francis Group; 2001. p. 3–17.

For peer review only

25. Conner M, Norman P. Predicting Health Behavior: Research and Practice with Social Cognition Models. 2005.

26. Rehm J. Measuring Quantity, Frequency, and Volume of Drinking. Alcohol Clin Exp Res. 1998;22(s2):4s–14s.

27. Shorter GW, Heather N, Bray JW, Giles EL, Holloway A, Barbosa C, et al. The 'Outcome Reporting in Brief Intervention Trials: Alcohol' (ORBITAL) framework: protocol to determine a core outcome set for efficacy and effectiveness trials of alcohol screening and brief intervention. Trials. 2017 Dec;18(1):611.

28. Shorter GW, Bray JW, Giles EL, O'Donnell AJ, Berman AH, Holloway A, et al. The Variability of Outcomes Used in Efficacy and Effectiveness Trials of Alcohol Brief Interventions: A Systematic Review. J Stud Alcohol Drugs. 2019 May;80(3):286–98.

29. Bendtsen M, Garnett C, Toner P, Shorter GW. The Effect of Question Order on Outcomes in the Core Outcome Set for Brief Alcohol Interventions Among Online Help-Seekers: Protocol for a Factorial Randomized Trial. JMIR Res Protoc. 2020 Nov 26;9(11):e24175.

30. SRNT Subcommittee on Biochemical Verification. Biochemical verification of tobacco use and cessation. Nicotine Tob Res. 2002 May;4(2):149–59.

31. Hays RD, Bjorner JB, Revicki DA, Spritzer KL, Cella D. Development of physical and mental health summary scores from the patient-reported outcomes measurement information system (PROMIS) global items. Qual Life Res. 2009 Sep;18(7):873–80.

32. Vallejo MA, Vallejo-Slocker L, Fernández-Abascal EG, Mañanes G. Determining Factors for Stress Perception Assessed with the Perceived Stress Scale (PSS-4) in Spanish and Other European Samples. Front Psychol. 2018 Jan 26;9.

33. Ajzen I. The theory of planned behavior. Organ Behav Hum Decis Process. 1991;50(2):179–211.

34. Bandura A. Self Efficacy: the exercise of control. Worth Publishers; 1997.

35. Rogers R. Cognitive and physiological processes in fear appeals and attitude change: A revised theory of protection motivation. In: Social Psychophysiological: A Sourcebook. 1983.

36. White IR, Royston P, Wood AM. Multiple imputation using chained equations: Issues and guidance for practice. Stat Med. 2011;30(4):377–99.

37. Bendtsen M. A Gentle Introduction to the Comparison Between Null Hypothesis Testing and Bayesian Analysis: Reanalysis of Two Randomized Controlled Trials. J Med Internet Res. 2018;20(10):e10873.

38. Bendtsen M. Electronic Screening for Alcohol Use and Brief Intervention by Email for University Students: Reanalysis of Findings From a Randomized Controlled Trial Using a Bayesian Framework. J Med Internet Res. 2019;21(11):e14419.

39. Bendtsen M. An Electronic Screening and Brief Intervention for Hazardous and Harmful Drinking Among Swedish University Students: Reanalysis of Findings From a Randomized Controlled Trial Using a Bayesian Framework. J Med Internet Res. 2019;21(12):e14420.

40. Imai K, Keele L, Tingley D. A General Approach to Causal Mediation Analysis. Psychol Methods. 2010;15(4):309–34.

41. Pearl J. Causality. Causality: Models, Reasoning, and Inference, Second Edition. Cambridge: Cambridge University Press; 2009.

42. Pearl J. Interpretation and identification of causal mediation. Psychol Methods. 2014;19(4):459–81.

For peer review only

For peer review only - http://bmjopen.bmj.com/site/about/guidelines.xhtml

43. Harrell F. Continuous Learning from Data: No Multiplicities from Computing and Using Bayesian Posterior Probabilities as Often as Desired [Internet]. 2020 [cited 2020 May 10]. Available from: https://www.fharrell.com/post/bayes-seq/

44. Norat T, Scoccianti C, Boutron-Ruault MC, Anderson A, Berrino F, Cecchini M, et al. European Code against Cancer 4th Edition: Diet and cancer. Cancer Epidemiol. 2015 Dec;39:S56–66.

45. Leon ME, Peruga A, McNeill A, Kralikova E, Guha N, Minozzi S, et al. European Code against Cancer, 4th Edition: Tobacco and cancer. Cancer Epidemiol. 2015 Dec;39:S20–33.

46. Scoccianti C, Cecchini M, Anderson AS, Berrino F, Boutron-Ruault MC, Espina C, et al. European Code against Cancer 4th Edition: Alcohol drinking and cancer. Cancer Epidemiol. 2016 Dec;45:181–8.

47. Noble N, Paul C, Turon H, Oldmeadow C. Which modifiable health risk behaviours are related? A systematic review of the clustering of Smoking, Nutrition, Alcohol and Physical activity ('SNAP') health risk factors. Prev Med. 2015 Dec;81:16–41.

48. Berrigan D, Dodd K, Troiano RP, Krebs-Smith SM, Barbash RB. Patterns of health behavior in U.S. adults. Prev Med. 2003 May;36(5):615–23.

49. Bendtsen M, Bendtsen P, Henriksson H, Henriksson P, Müssener U, Thomas K, et al. The Mobile Health Multiple Lifestyle Behavior Interventions Across the Lifespan (MoBILE) Research Program: Protocol for Development, Evaluation, and Implementation. JMIR Res Protoc. 2020;9(4):e14894.

50. Meader N, King K, Wright K, Graham HM, Petticrew M, Power C, et al. Multiple Risk Behavior Interventions: Meta-analyses of RCTs. Am J Prev Med. 2017;53(1):e19–30.

51. Prochaska JJ, Spring B, Nigg CR. Multiple health behavior change research: An introduction and overview. Prev Med. 2008;46(3):181–8.

52. De Vries H, Kremers S, Smeets T, Reubsaet A. Clustering of diet, physical activity and smoking and a general willingness to change. Psychol Health. 2008;23(3):265–78.

53. Alageel S, Gulliford MC, McDermott L, Wright AJ. Multiple health behaviour change interventions for primary prevention of cardiovascular disease in primary care: systematic review and meta-analysis. BMJ Open. 2017 Jun;7(6):e015375.

54. Perski O, Watson NL, Mull KE, Bricker JB. Identifying Content-Based Engagement Patterns in a Smoking Cessation Website and Associations With User Characteristics and Cessation Outcomes: A Sequence and Cluster Analysis. Nicotine Tob Res. 2021 Jun 8;23(7):1103–12.

55. McCambridge J, Kypri K, Elbourne D. Research participation effects: a skeleton in the methodological cupboard. J Clin Epidemiol. 2014 Aug;67(8):845–9.

56. McCambridge J, Witton J, Elbourne DR. Systematic review of the Hawthorne effect: New concepts are needed to study research participation effects. J Clin Epidemiol. 2014 Mar;67(3):267–77.

57. Higgins JPT, Altman DG, Gotzsche PC, Juni P, Moher D, Oxman AD, et al. The Cochrane Collaboration's tool for assessing risk of bias in randomised trials. BMJ. 2011 Oct 18;343(oct18 2):d5928–d5928.

58. Bendtsen M, McCambridge J, Åsberg K, Bendtsen P. Text Messaging Interventions for Reducing Alcohol Consumption Among risky drinkers: Systematic Review and Meta-Analysis. Addiction. 2020;

59. Miles LM, Elbourne D, Farmer A, Gulliford M, Locock L, McCambridge J, et al. Bias due to MEasurement Reactions In Trials to improve health (MERIT): protocol for research to develop MRC guidance. Trials. 2018 Dec 26;19(1):653.

60. McCambridge J. From question-behaviour effects in trials to the social psychology of research participation. Psychol Health. 2015 Jan 2;30(1):72–84.

For peer review only

61. Hrobjartsson A, Thomsen ASS, Emanuelsson F, Tendal B, Hilden J, Boutron I, et al. Observer bias in randomised clinical trials with binary outcomes: systematic review of trials with both blinded and non-blinded outcome assessors. BMJ. 2012 Feb 27;344(feb27 2):e1119–e1119.

FIGURE LEGENDS

Figure 1 - SPIRIT figure depicting participant timeline.

For peer review only

	STUDY PERIOD					
	**Enrolment**	**Allocation**	**Post-allocation**			**Close-out**
**TIMEPOINT**	**0**	**0**	**0**	**1 month**	**2 months**	**4 months**
**ENROLMENT:**						
**Informed consent**	X					
**Eligibility screen**	X					
**Allocation**		X				
**INTERVENTIONS:**						
Digital intervention (factorial design)		X	◆———	———	———	———◆
**ASSESSMENTS:**						
Baseline questionnaire	X					
Mediator questionnaire	X			X	X	X
Lifestyle outcomes questionnaire					X	X
Perceived stress	X				X	X
QoL						X
Participant experience						X

For peer review only

APPENDIX A – QUESTIONNAIRES

Note: Participants are reminded of the definition of a standard drink of alcohol by graphical means, as well as given visual cues for what constitutes a portion of fruit and vegetables.

BASELINE QUESTIONNAIRE

1. Sex:
   a. Female
   b. Male

2. Age (numerical measure)

3. How many standard drinks of alcohol did you consume last week? (numerical measure)

4. How often, during the past month, have you consumed four/five (female/male) or more standard drinks of alcohol on one occasion? (numerical measure)

5. How many cigarettes did you smoke last week? (numerical measure)

6. How much time in total did you spend on moderate physical activity (e.g. bicycling or walking for transport or leisure) **last week**?
   a. 0
   b. Less than 30 minutes
   c. 30-60 minutes
   d. 1 hours
   e. 1.5 hours
   f. 2 hours
   g. 2.5 hours
   h. 3 hours
   i. 3.5 hours (i.e. 30 minutes per day)
   j. 4 hours
   k. 5 hours
   l. 6 hours
   m. 7 hours (i.e. 1 hour per day)
   n. 10.5 hours (i.e. 1.5 hours per day)
   o. 14 hours (i.e. 2 hours per day)

7. How much time in total did you spend on vigorous physical activity (i.e. producing increases in breathing or heart rate), for instance running, aerobics, etc. **last week?**
   a. 0
   b. Less than 30 minutes
   c. 30-60 minutes
   d. 1 hours
   e. 1.5 hours
   f. 2 hours
   g. 2.5 hours
   h. 3 hours
   i. 3.5 hours (i.e. 30 minutes per day)

For peer review only

j. 4 hours
k. 5 hours
l. 6 hours
m. 7 hours (i.e. 1 hour per day)
n. 10.5 hours (i.e. 1.5 hours per day)
o. 14 hours (i.e. 2 hours per day)

8. How many 100g portions (equivalent to an average sized banana or one large apple) of fruit did you consume **last week**?
   a. 0
   b. 1-2 portions **per week**
   c. 3-4 portions **per week**
   d. 5-6 portion **per week**
   e. 1.0 portion **per day**
   f. 1.5 portions **per day**
   g. 2.0 portions **per day**
   h. 2.5 portions **per day**
   i. 3.0 portions **per day or more**

9. How many 100 g portions (equivalent to an average handful) of vegetables did you consume **last week**?
   a. 0
   b. 1-2 portions **per week**
   c. 3-4 portions **per week**
   d. 5-6 portion **per week**
   e. 1.0 portion **per day**
   f. 1.5 portions **per day**
   g. 2.0 portions **per day**
   h. 2.5 portions **per day**
   i. 3.0 portions **per day or more**

10. In the last month, how often have you felt that you were unable to control the important things in your life?
    a. Never
    b. Almost never
    c. Sometimes
    d. Fairly often
    e. Very often

11. In the last month, how often have you felt confident about your ability to handle your personal problems?
    a. Never
    b. Almost never
    c. Sometimes
    d. Fairly often
    e. Very often

12. In the last month, how often have you felt that things were going your way?
    a. Never
    b. Almost never

For peer review only

c. Sometimes
d. Fairly often
e. Very often

13. In the last month, how often have you felt difficulties were piling up so high that you could not overcome them?
    a. Never
    b. Almost never
    c. Sometimes
    d. Fairly often
    e. Very often

14. How important is it for you to improve your lifestyle behaviours? (10-point scale ranging from 1 = "Not important" to 10 = "Very important")

15. How confident are you that you will be able to improve your lifestyle behaviours? (10-point scale ranging from 1 = "Not at all" to 10 = "Very confident")

16. To what degree do you have the know-how and strategies to improve your lifestyle behaviours? (10-point scale ranging from 1 = "Not at all" to 10 = "Very high degree")

1-MONTH FOLLOW-UP (MEDIATORS ONLY) QUESTIONNAIRE

1. How important is it for you to improve your lifestyle behaviours? (10-point scale ranging from 1 = "Not important" to 10 = "Very important")

2. How confident are you that you will be able to improve your lifestyle behaviours? (10-point scale ranging from 1 = "Not at all" to 10 = "Very confident")

3. To what degree do you have the know-how and strategies to improve your lifestyle behaviours? (10-point scale ranging from 1 = "Not at all" to 10 = "Very high degree")

2- AND 4-MONTH FOLLOW-UP QUESTIONNAIRE

1. How many standard drinks of alcohol did you consume last week? (numerical measure)

2. How often, during the past month, have you consumed four/five (female/male) or more standard drinks of alcohol on one occasion? (numerical measure)

3. Have you smoked any cigarettes the past four weeks?
    a. Yes
    b. No

4. (Smokers only) How many cigarettes did you smoke last week? (numerical measure)

5. How much time in total did you spend on moderate physical activity (e.g. bicycling or walking for transport or leisure) **last week**?
    a. 0
    b. Less than 30 minutes
    c. 30-60 minutes
    d. 1 hours

For peer review only

e. 1.5 hours
f. 2 hours
g. 2.5 hours
h. 3 hours
i. 3.5 hours (i.e. 30 minutes per day)
j. 4 hours
k. 5 hours
l. 6 hours
m. 7 hours (i.e. 1 hour per day)
n. 10.5 hours (i.e. 1.5 hours per day)
o. 14 hours (i.e. 2 hours per day)

6. How much time in total did you spend on vigorous physical activity (i.e. producing increases in breathing or heart rate), for instance running, aerobics, etc. **last week?**
   a. 0
   b. Less than 30 minutes
   c. 30-60 minutes
   d. 1 hours
   e. 1.5 hours
   f. 2 hours
   g. 2.5 hours
   h. 3 hours
   i. 3.5 hours (i.e. 30 minutes per day)
   j. 4 hours
   k. 5 hours
   l. 6 hours
   m. 7 hours (i.e. 1 hour per day)
   n. 10.5 hours (i.e. 1.5 hours per day)
   o. 14 hours (i.e. 2 hours per day)

7. How many 100g portions (equivalent to an average sized banana or one large apple) of fruit did you consume **last week**?
   a. 0
   b. 1-2 portions **per week**
   c. 3-4 portions **per week**
   d. 5-6 portion **per week**
   e. 1.0 portion **per day**
   f. 1.5 portions **per day**
   g. 2.0 portions **per day**
   h. 2.5 portions **per day**
   i. 3.0 portions **per day or more**

8. How many 100 g portions (equivalent to an average handful) of vegetables did you consume **last week**?
   a. 0
   b. 1-2 portions **per week**
   c. 3-4 portions **per week**
   d. 5-6 portion **per week**
   e. 1.0 portion **per day**
   f. 1.5 portions **per day**

For peer review only

g. 2.0 portions **per day**
h. 2.5 portions **per day**
i. 3.0 portions **per day or more**

9. How many cans (33 cl, one standard can) of sugary drinks (e.g. soft/fizzy drinks, "energy drinks") did you consume **last week**?
    a. 0 cans
    b. 1 can **per week**
    c. 2-3 cans **per week**
    d. 4-6 cans **per week**
    e. 1 can **per day**
    f. 1.5 cans **per day**
    g. 2.0 cans **per day**
    h. 2.5 cans **per day**
    i. 3.0 cans **per day or more**

10. How many portions of sweets, chocolate, pastry (e.g. buns, muffins, biscuits), ice cream and salty snacks (e.g. crisps, nuts, cheese doodles) did you eat **last week**? *One portion is 50 g sweets (9 pieces), 40 g chocolate (6 pieces/squares), 1 bun, 2 dl (scoops) of ice cream or 2 dl snacks (40 g).*

    a. 0 portions
    b. 1 portion **per week**
    c. 2-3 portions **per week**
    d. 4-6 portions **per week**
    e. 1 portion **per day**
    f. 1.5 portions **per day**
    g. 2.0 portions **per day**
    h. 2.5 portions **per day**
    i. 3.0 portions **per day**
    j. 3.5 portions **per day**
    k. 4.0 portions **per day or more**

11. How tall are you? (numerical measure)

12. What is your current body weight? (numerical measure)

13. In the last month, how often have you felt that you were unable to control the important things in your life?
    a. Never
    b. Almost never
    c. Sometimes
    d. Fairly often
    e. Very often

14. In the last month, how often have you felt confident about your ability to handle your personal problems?
    a. Never
    b. Almost never
    c. Sometimes

For peer review only

   d. Fairly often
   e. Very often

15. In the last month, how often have you felt that things were going your way?
   a. Never
   b. Almost never
   c. Sometimes
   d. Fairly often
   e. Very often

16. In the last month, how often have you felt difficulties were piling up so high that you could not overcome them?
   a. Never
   b. Almost never
   c. Sometimes
   d. Fairly often
   e. Very often

17. How important is it for you to improve or maintain healthy lifestyle behaviours? (10-point scale ranging from 1 = "Not important" to 10 = "Very important")

18. How confident are you that you will be able to improve or maintain healthy lifestyle behaviours? (10-point scale ranging from 1 = "Not at all" to 10 = "Very confident")

19. To what degree do you have the know-how and strategies to improve or maintain healthy lifestyle behaviours? (10-point scale ranging from 1 = "Not at all" to 10 = "Very high degree")

4-MONTH FOLLOW-UP ONLY

1. Overall, how well do you believe that the support given to you suited your needs?
   a. I feel like I did not receive any support at all
   b. I feel like I received some support, but it did not suit my needs
   c. I feel like I received some support, and it did suit my needs
   d. I feel like I received all the support that I needed

2. (If a or b to question 2): You have responded that you did not receive adequate support, what did you do instead?
   a. I decided to find other ways to help me change my lifestyle
   b. I decided to not make any change to my lifestyle
   c. Other (please comment)

3. Please leave a comment describing your needs and how the support did or did not address them (Free-text).

4. Do you believe that the support given to you would be helpful for other individuals that want to change their lifestyle? (1 = "Not very helpful" to 5 = "Very helpful")

5. Would you recommend the support you were given to a friend who expresses a wish to change their lifestyle?
   a. Yes

For peer review only

   b. No
   c. I do not know

6. If you were to continue using the support, for how much longer would you want to use it?
   a. I would use it for one to two more months
   b. I would use it for three to six more months
   c. I would use it for more than six months
   d. I would not use it any more
   e. I do not know

7. In general, would you say your health is: (Poor, Fair, Good, Very good, Excellent)

8. In general, would you say your quality of life is: (Poor, Fair, Good, Very good, Excellent)

9. In general, how would you rate your physical health: (Poor, Fair, Good, Very good, Excellent)

10. In general, how would you rate your mental health, including your mood and your ability to think? (Poor, Fair, Good, Very good, Excellent)

11. In general, how would you rate your satisfaction with your social activities and relationships? (Poor, Fair, Good, Very good, Excellent)

12. In general, please rate how well you carry out your usual social activities. This includes activities at home, at work and in your community, and responsibilities as a parent, child, spouse, employee, friend, etc.: (Poor, Fair, Good, Very good, Excellent)

13. To what extent are you able to carry out your everyday physical activities such as walking, climbing stairs, carrying groceries, or moving a chair?
    a. Not at all
    b. A little
    c. Moderately
    d. Mostly
    e. Completely

14. In the past 7 days, how often have you been bothered by emotional problems such as feeling anxious depressed or irritable?
    a. Always
    b. Often
    c. Sometimes
    d. Rarely
    e. Never

15. In the past 7 days, how would you rate your fatigue on average?
    a. Very severe
    b. Severe
    c. Moderate
    d. Mild
    e. None

For peer review only

16. In the past 7 days, how would you rate your pain on average (where 0 is No Pain, and 10 is Worst Pain Imaginable)?

For peer review only

APPENDIX B – INTERVENTION DESCRIPTION AND FACTORIAL CONDITIONS

This appendix describes the content of the *Coach* intervention, which is based on an intervention design we have used previously [1,2]. The intervention targets alcohol, diet, physical activity, and smoking. The factorial conditions of the trial are also explained in detail in this appendix.

The intervention was developed with inspiration from the first four steps of the Intervention Mapping (IM) approach [3]. The intervention is based on social cognitive models for behaviour change where environment, intentions and skills often are highlighted as important for change [4,5]. Therefore, we identified and designed components which intended to affect these factors. This was based on our previous research in Sweden [1,6–12] and the research literature more widely (see specific descriptions below). The logic model in Supplementary Appendix Figure 1 gives an overview of the reasoning behind the intervention, including outcomes and potential short-, mid- and long-term impact.

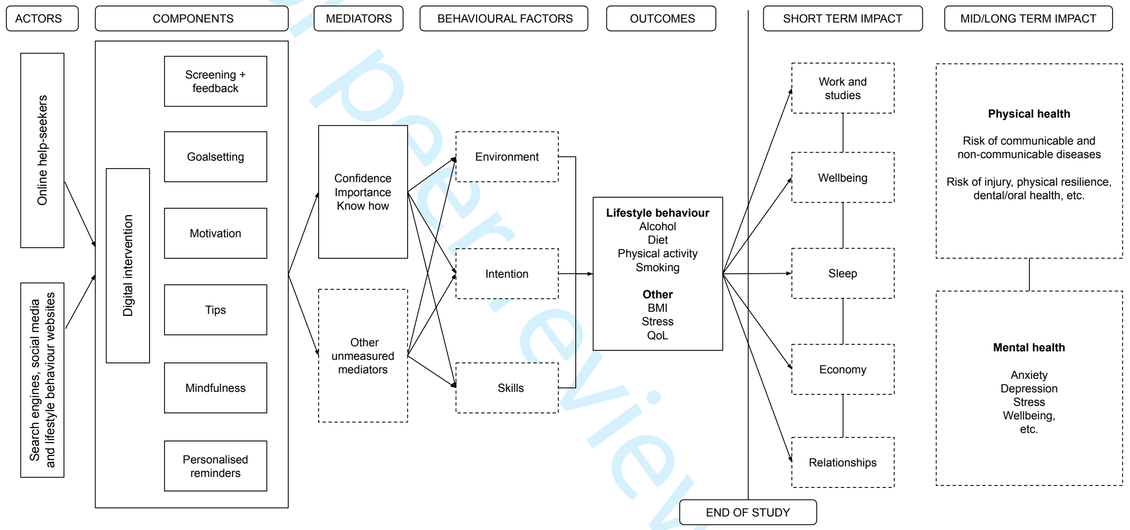

Supplementary Appendix Figure 1 - Logic model showing actors, intervention components, mediators, behavioural factors, outcomes, and short-, mid- and long-term impacts

INTERVENTION DESIGN AND COMPONENTS

The intervention is intended to be used as a toolbox, enabling users to decide which intervention content they want to interact with and when. As can be seen in the screenshot in Supplementary Appendix Figure 2, the design of the intervention allows for each component to be presented to participants in a menu, allowing easy addition and removal of components based on factorial condition. Follows does a description of each component, using the BCTTv1 93-item taxonomy [13] to specify techniques included when appropriate.

For peer review only

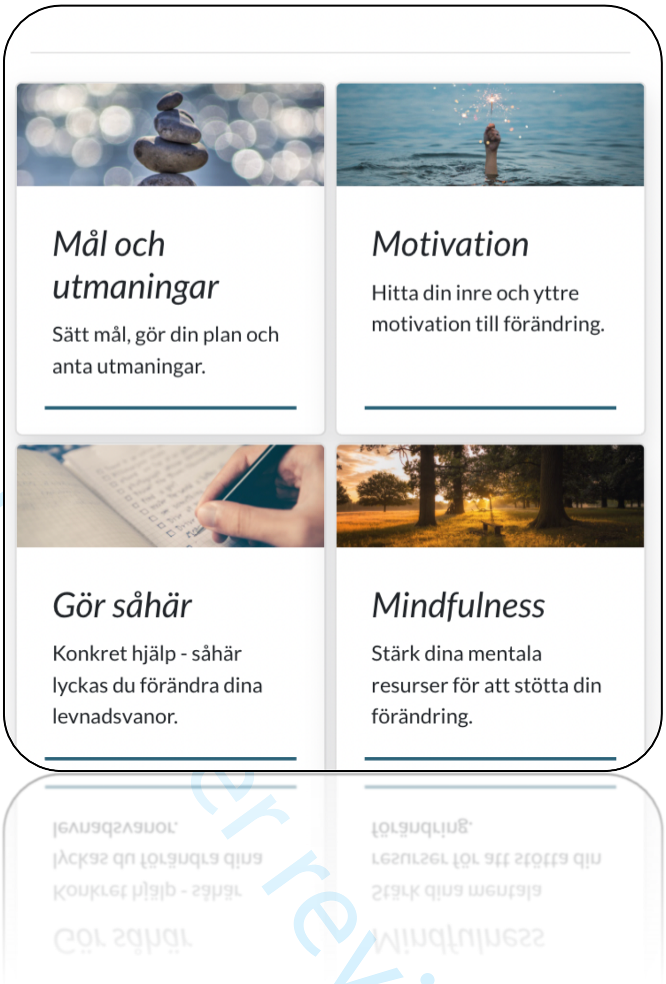

Supplementary Appendix Figure 2 - A screenshot of the digital intervention showing the main menu

COMPONENT 1: SCREENING AND FEEDBACK

The first component consists of screening and feedback. Every Sunday afternoon, participants will receive a text message with a hyperlink. When pressing the link, participants will be asked to respond to a questionnaire regarding their current lifestyle behaviours, after which they are shown feedback on their current behaviour in contrast to national guidelines (see screenshots in Supplementary Appendix Figure 3). They will subsequently be given access to the rest of the components appropriate for their randomised allocation. Self-monitoring has been shown to be a potentially effective strategy for reducing excessive alcohol consumption [14–17] and to promote healthy eating and physical activity [18,19]. When this component is absent, participants will not be asked to respond to the screening questionnaire but will instead be shown national guidelines without any feedback. BCTs used: Discrepancy between current behaviour and goal (BCT 1.6), Feedback on behaviour (BCT 2.2), Self-monitoring of behaviour (BCT 2.3), and Social comparison (BCT 6.2).

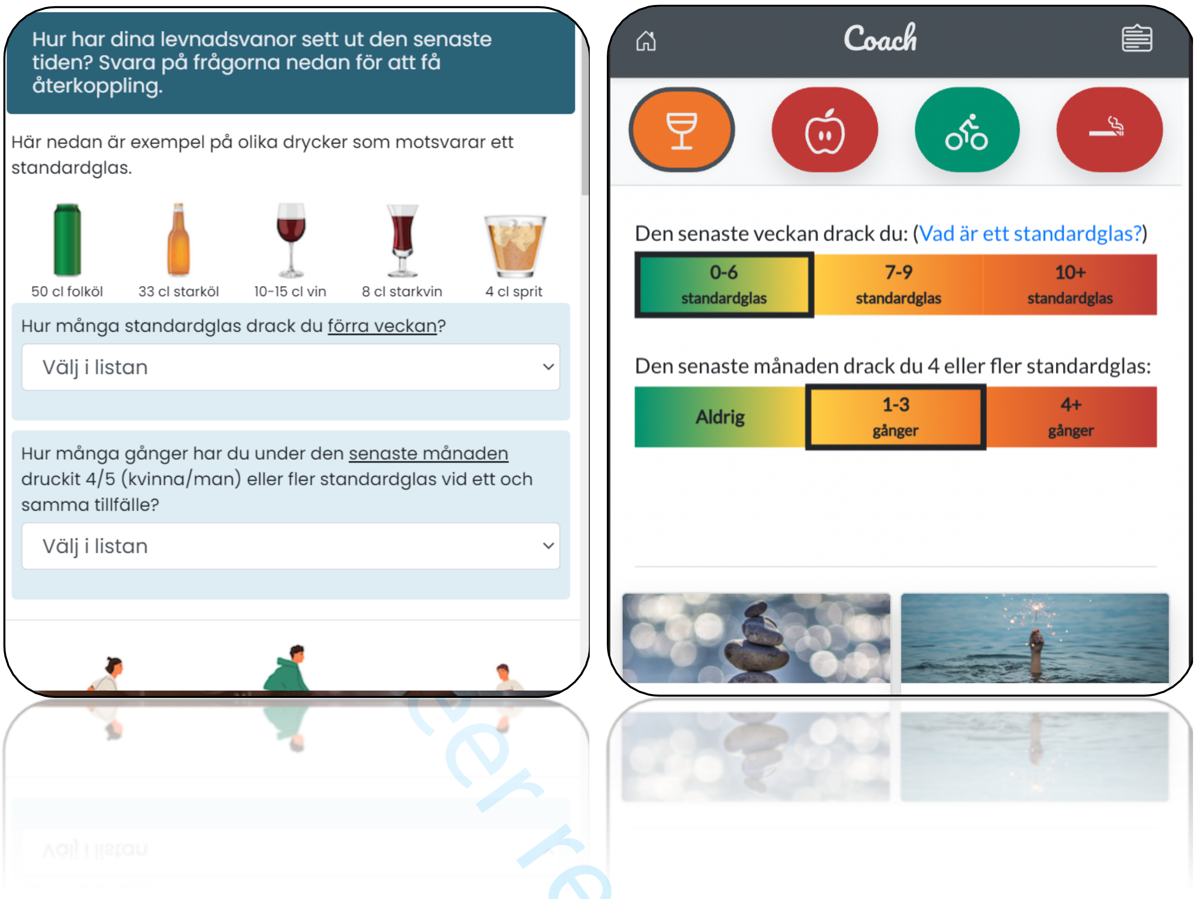

Supplementary Appendix Figure 3 – Screenshots of the digital intervention showing screening and feedback based on national guidelines

COMPONENT 2: GOALSETTING AND PLANNING

The second component supports enhanced self-regulatory capacity and skills via goalsetting and planning. This includes setting goals for future behaviour, preparing for triggers, and accepting both custom and ready-made challenges. Intervention content designed around goalsetting, action planning, practicing behaviour, and habit formation have, amongst other planning related activities, been shown to be important among effective lifestyle interventions [18,20–25]. Participants will be reminded of the goals that they have set, including any challenges they have accepted, via text message prompts throughout the week (up to 4 messages). BCTs used: Goal setting (behaviour) (BCT 1.1), Problem solving (BCT 1.2), Action planning (BCT 1.4), Prompts/cues (BCT 7.1), Behaviour practice/rehearsal (BCT 8.1), Behaviour substitution (BCT 8.2), Habit formation (BCT 8.3), Graded tasks (BCT 8.7).

COMPONENT 3: MOTIVATION

The third component aims to increase users' awareness of their own motivation, prompt commitment, and boost motivation. This is supported via texts, videos and exercises relating to health, economics, and motivation awareness. Digital behaviour change interventions have been shown to have the capacity to increase self-efficacy, however, there is lack of consensus across reviews with regards to which content works to facilitate an increase of self-efficacy [26]. The component will also allow participants to sign up for text messages with motivational content sent to them throughout the week. Participants choose which behaviours

For peer review only - http://bmjopen.bmj.com/site/about/guidelines.xhtml

they wish to have messages for, with a maximum of 8-10 messages per week. The content of the messages has been derived from previously developed and evaluated interventions [6–12]. BCTs used: Information about health consequences (BCT 5.1), Credible source (9.1), Pros and cons (BCT 9.2), Comparative imagining of future outcomes (BCT 9.3).

COMPONENT 4: SKILLS AND KNOW-HOW

The fourth component aims to increase user's skills and know-how of how to make lasting behavioural changes. This will include concrete tips on how to initiate and maintain change in everyday life. For instance, participants are given strategies they can employ when going to parties where alcohol is served, or how to introduce vegetables to their meals. As with the third component, participants will be able to sign up for text messages with tips sent to them throughout the week (maximum 8-10 per week) – the content of which has also been derived from previously developed and evaluated interventions [6–12]. BCTs used: Social support (unspecified) (BCT 3.1), Instructions on how to perform a behaviour (BCT 4.1), Self-incentive (BCT 10.7), and Self-reward (BCT 10.9).

COMPONENT 5: MINDFULNESS

The fifth component aims to increase users' awareness of their own lived experience and strengthen their capacity for a non-reactive, compassionate, and less stressful way of being in the world. The practices thus help participants to build the mental resources needed for behaviour change. A set of mindfulness exercises, including guided meditations, will be available in the component. The exercises are based on previous research, and are considered evidence-based methods to improve the mental well-being of clinical populations, while effects in non-clinical settings and behaviour change are less studied [27–31].

COMPONENT 6: SELF-COMPOSED TEXT MESSAGES

The sixth component consists of self-composed text messages sent to participants throughout the week. Participants will be allowed to author up to three messages to themselves and have them sent at specified intervals. For instance, a participant can write a message about their commitment to increase their physical activity and decide to have it sent to them every Monday and Wednesday at 5pm. This type of activity seems generally under-studied in the literature, but has shown preliminary interesting results in an ongoing trial [32].

For peer review only

FACTORIAL ALLOCATIONS

Using P to represent *present* and A to represent *absent*, the 64 factorial conditions are presented in Supplementary Appendix Table 1. As is evident from the table, each component will be available to half the study population, allowing for contrasts between *present* and *absent* to be fully powered by the sample. However, as one is estimating the effects of individual components, effect sizes may be smaller than when contrasting the full intervention versus a control, which may increase the sample size required.

Supplementary Appendix Table 1 - Combination of components in 64 factorial conditions (P = present , A = absent)

Conditions	Component 1	Component 2	Component 3	Component 4	Component 5	Component 6
1	P	P	P	P	P	P
2	P	P	P	P	P	A
3	P	P	P	P	A	P
4	P	P	P	P	A	A
5	P	P	P	A	P	P
6	P	P	P	A	P	A
7	P	P	P	A	A	P
8	P	P	P	A	A	A
9	P	P	A	P	P	P
10	P	P	A	P	P	A
11	P	P	A	P	A	P
12	P	P	A	P	A	A
13	P	P	A	A	P	P
14	P	P	A	A	P	A
15	P	P	A	A	A	P
16	P	P	A	A	A	A
17	P	A	P	P	P	P
18	P	A	P	P	P	A
19	P	A	P	P	A	P
20	P	A	P	P	A	A
21	P	A	P	A	P	P
22	P	A	P	A	P	A
23	P	A	P	A	A	P
24	P	A	P	A	A	A
25	P	A	A	P	P	P
26	P	A	A	P	P	A
27	P	A	A	P	A	P
28	P	A	A	P	A	A
29	P	A	A	A	P	P
30	P	A	A	A	P	A
31	P	A	A	A	A	P
32	P	A	A	A	A	A
33	A	P	P	P	P	P

For peer review only

34	A	P	P	P	P	A
35	A	P	P	P	A	P
36	A	P	P	P	A	A
37	A	P	P	A	P	P
38	A	P	P	A	P	A
39	A	P	P	A	A	P
40	A	P	P	A	A	A
41	A	P	A	P	P	P
42	A	P	A	P	P	A
43	A	P	A	P	A	P
44	A	P	A	P	A	A
45	A	P	A	A	P	P
46	A	P	A	A	P	A
47	A	P	A	A	A	P
48	A	P	A	A	A	A
49	A	A	P	P	P	P
50	A	A	P	P	P	A
51	A	A	P	P	A	P
52	A	A	P	P	A	A
53	A	A	P	A	P	P
54	A	A	P	A	P	A
55	A	A	P	A	A	P
56	A	A	P	A	A	A
57	A	A	A	P	P	P
58	A	A	A	P	P	A
59	A	A	A	P	A	P
60	A	A	A	P	A	A
61	A	A	A	A	P	P
62	A	A	A	A	P	A
63	A	A	A	A	A	P
64	A	A	A	A	A	A

For peer review only

REFERENCES

1. Bendtsen M, McCambridge J. Reducing alcohol consumption among risky drinkers in the general population of Sweden using an interactive mobile health intervention: protocol for a randomized controlled trial. JMIR Research Protocols. 2019;8(4):e13119.

2. Åsberg K, Lundgren O, Henriksson H, Henriksson P, Bendtsen P, Löf M, et al. Multiple lifestyle behaviour mHealth intervention targeting Swedish college and university students: protocol for the *Buddy* randomised factorial trial. BMJ Open. 2021 Dec;11(12):e051044.

3. Bartholomew Eldredge LK. Planning health promotion programs: an intervention mapping approach. Fourth edition. San Francisco, CA: Jossey-Bass & Pfeiffer Imprints, Wiley; 2016. 1 p.

4. Fishbein M, Triandis HC, Kanfer FH, Becker M, Middlestadt SE, Eichler A. Factors influencing behaviour and behaviour change. In: Handbook of Health Psychology. Psychology Press Taylor & Francis Group; 2001. p. 3–17.

5. Conner M, Norman P. Predicting health behaviour: research and practice with social cognition models. Open University Press; 2005.

6. Müssener U, Bendtsen M, Karlsson N, White IR, McCambridge J, Bendtsen P. Effectiveness of Short Message Service Text-Based Smoking Cessation Intervention Among University Students. JAMA Internal Medicine. 2016;176(3):321.

7. Müssener U, Bendtsen M, Karlsson N, White IR, McCambridge J, Bendtsen P. SMS-based smoking cessation intervention among university students: study protocol for a randomised controlled trial (NEXit trial). Trials. 2015;16(1):140.

8. Thomas K, Bendtsen M, Linderoth C, Karlsson N, Bendtsen P, Müssener U. Short message service (SMS)-based intervention targeting alcohol consumption among university students: study protocol of a randomized controlled trial. Trials. 2017;18(1):156.

9. Thomas K, Müssener U, Linderoth C, Karlsson N, Bendtsen P, Bendtsen M. Effectiveness of a Text Messaging–Based Intervention Targeting Alcohol Consumption Among University Students: Randomized Controlled Trial. JMIR mHealth and uHealth. 2018;6(6):e146.

10. Müssener U, Bendtsen M, McCambridge J, Bendtsen P. User satisfaction with the structure and content of the NEXit intervention, a text messaging-based smoking cessation programme. BMC Public Health. 2016;16(1):1179.

11. Mussener U, Thomas K, Linderoth C, Leijon M, Bendtsen M. A Text Message-Based Intervention Targeting Alcohol Consumption Among University Students: User Satisfaction and Acceptability Study. JMIR human factors. 2018;5(3):e23.

12. Thomas K, Linderoth C, Bendtsen M, Bendtsen P, Mussener U. Text Message-Based Intervention Targeting Alcohol Consumption Among University Students: Findings From a Formative Development Study. JMIR mHealth and uHealth. 2016;4(4):e119.

13. Michie S, Richardson M, Johnston M, Abraham C, Francis J, Hardeman W, et al. The Behavior Change Technique Taxonomy (v1) of 93 Hierarchically Clustered Techniques: Building an International Consensus for the Reporting of Behavior Change Interventions. Annals of Behavioral Medicine. 2013 Aug;46(1):81–95.

14. Michie S, Whittington C, Hamoudi Z, Zarnani F, Tober G, West R. Identification of behaviour change techniques to reduce excessive alcohol consumption. Addiction (Abingdon, England). 2012 Aug;107(8):1431–40.

15. Bendtsen P, McCambridge J, Bendtsen M, Karlsson N, Nilsen P. Effectiveness of a Proactive Mail-Based Alcohol Internet Intervention for University Students: Dismantling the Assessment and Feedback Components in a Randomized Controlled Trial. Journal of Medical Internet Research. 2012 Oct 31;14(5):e142.

16. McCambridge J, Bendtsen M, Karlsson N, White IR, Nilsen P, Bendtsen P. Alcohol assessment and feedback by email for university students: Main findings from a randomised controlled trial. British Journal of Psychiatry. 2013;203(5):334–40.

17. Bendtsen P, Bendtsen M, Karlsson N, White IR, McCambridge J. Online Alcohol Assessment and Feedback for Hazardous and Harmful Drinkers: Findings From the AMADEUS-2 Randomized Controlled Trial of Routine Practice in Swedish Universities. Journal of Medical Internet Research. 2015;17(7):e170.

For peer review only

18. Michie S, Abraham C, Whittington C, Mcateer J. Effective Techniques in Healthy Eating and Physical Activity Interventions : A Meta-Regression. 2009;28(6):690–701.

19. Murray JM, Brennan SF, French DP, Patterson CC, Kee F, Hunter RF. Effectiveness of physical activity interventions in achieving behaviour change maintenance in young and middle aged adults: A systematic review and meta-analysis. Social science & medicine (1982). 2017 Nov;192:125–33.

20. Knittle K, Nurmi J, Crutzen R, Hankonen N, Beattie M, Dombrowski SU. How can interventions increase motivation for physical activity? A systematic review and meta-analysis. Health psychology review. 2018 Sep;12(3):211–30.

21. Howlett N, Trivedi D, Troop NA, Chater AM. Are physical activity interventions for healthy inactive adults effective in promoting behavior change and maintenance, and which behavior change techniques are effective? A systematic review and meta-analysis. Translational behavioral medicine. 2019 Jan;9(1):147–57.

22. Ashton LM, Sharkey T, Whatnall MC, Williams RL, Bezzina A, Aguiar EJ, et al. Effectiveness of Interventions and Behaviour Change Techniques for Improving Dietary Intake in Young Adults: A Systematic Review and Meta-Analysis of RCTs. Nutrients. 2019 Apr;11(4).

23. Garnett C V, Crane D, Brown J, Kaner EFS, Beyer FR, Muirhead CR, et al. Behavior Change Techniques Used in Digital Behavior Change Interventions to Reduce Excessive Alcohol Consumption: A Meta-regression. Annals of behavioral medicine : a publication of the Society of Behavioral Medicine. 2018 May;52(6):530–43.

24. McCrabb S, Baker AL, Attia J, Skelton E, Twyman L, Palazzi K, et al. Internet-Based Programs Incorporating Behavior Change Techniques Are Associated With Increased Smoking Cessation in the General Population: A Systematic Review and Meta-analysis. Annals of behavioral medicine : a publication of the Society of Behavioral Medicine. 2019 Feb;53(2):180–95.

25. Ek A, Alexandrou C, Söderström E, Bergman P, Delisle Nyström C, Direito A, et al. Effectiveness of a 3-month mobile phone based behavior change program on active transportation and physical activity in adults: A randomized controlled trial. JMIR mHealth uHealth. 2020;8(6).

26. Newby K, Teah G, Cooke R, Li X, Brown K, Salisbury-Finch B, et al. Do automated digital health behaviour change interventions have a positive effect on self-efficacy? A systematic review and meta-analysis. Health Psychology Review. 2020 Jan 20;1–19.

27. Crane RS, Brewer J, Feldman C, Kabat-Zinn J, Santorelli S, Williams JMG, et al. What defines mindfulness-based programs? The warp and the weft. Psychological Medicine. 2017 Apr 29;47(6):990–9.

28. Creswell JD. Mindfulness Interventions. Annual Review of Psychology. 2017 Jan 3;68(1):491–516.

29. Wong SYS, Chan JYC, Zhang D, Lee EKP, Tsoi KKF. The Safety of Mindfulness-Based Interventions: a Systematic Review of Randomized Controlled Trials. Mindfulness. 2018 Oct 2;9(5):1344–57.

30. Galante J, Friedrich C, Dawson AF, Modrego-Alarcón M, Gebbing P, Delgado-Suárez I, et al. Mindfulness-based programmes for mental health promotion in adults in nonclinical settings: A systematic review and meta-analysis of randomised controlled trials. Patel V, editor. PLOS Medicine. 2021 Jan 11;18(1):e1003481.

31. Neff KD, Germer CK. A Pilot Study and Randomized Controlled Trial of the Mindful Self-Compassion Program. Journal of Clinical Psychology. 2013 Jan;69(1):28–44.

32. Bendtsen M, McCambridge J. Reducing Alcohol Consumption Among Risky Drinkers in the General Population of Sweden Using an Interactive Mobile Health Intervention: Protocol for a Randomized Controlled Trial. JMIR Research Protocols. 2019;8(4):e13119.

For peer review only

For peer review only

STANDARD PROTOCOL ITEMS: RECOMMENDATIONS FOR INTERVENTIONAL TRIALS

SPIRIT 2013 Checklist: Recommended items to address in a clinical trial protocol and related documents*

Section/item	Item No	Description	Addressed on page number
**Administrative information**			
Title	1	Descriptive title identifying the study design, population, interventions, and, if applicable, trial acronym	______1______
Trial registration	2a	Trial identifier and registry name. If not yet registered, name of intended registry	_____1,2______
	2b	All items from the World Health Organization Trial Registration Data Set	_____1,2______
Protocol version	3	Date and version identifier	______NA____
Funding	4	Sources and types of financial, material, and other support	_____ 14_____
Roles and responsibilities	5a	Names, affiliations, and roles of protocol contributors	_____ 1______
	5b	Name and contact information for the trial sponsor	_____14____
	5c	Role of study sponsor and funders, if any, in study design; collection, management, analysis, and interpretation of data; writing of the report; and the decision to submit the report for publication, including whether they will have ultimate authority over any of these activities	_____14____
	5d	Composition, roles, and responsibilities of the coordinating centre, steering committee, endpoint adjudication committee, data management team, and other individuals or groups overseeing the trial, if applicable (see Item 21a for data monitoring committee)	_____NA_____

For peer review only

Introduction

Background and rationale	6a	Description of research question and justification for undertaking the trial, including summary of relevant studies (published and unpublished) examining benefits and harms for each intervention	_____3______
	6b	Explanation for choice of comparators	_____3_______
Objectives	7	Specific objectives or hypotheses	_____3_______
Trial design	8	Description of trial design including type of trial (eg, parallel group, crossover, factorial, single group), allocation ratio, and framework (eg, superiority, equivalence, noninferiority, exploratory)	_____4______

Methods: Participants, interventions, and outcomes

Study setting	9	Description of study settings (eg, community clinic, academic hospital) and list of countries where data will be collected. Reference to where list of study sites can be obtained	_____4_______
Eligibility criteria	10	Inclusion and exclusion criteria for participants. If applicable, eligibility criteria for study centres and individuals who will perform the interventions (eg, surgeons, psychotherapists)	_____4______
Interventions	11a	Interventions for each group with sufficient detail to allow replication, including how and when they will be administered	____4,5_______
	11b	Criteria for discontinuing or modifying allocated interventions for a given trial participant (eg, drug dose change in response to harms, participant request, or improving/worsening disease)	_____NA______
	11c	Strategies to improve adherence to intervention protocols, and any procedures for monitoring adherence (eg, drug tablet return, laboratory tests)	_____NA_____
	11d	Relevant concomitant care and interventions that are permitted or prohibited during the trial	_____NA______
Outcomes	12	Primary, secondary, and other outcomes, including the specific measurement variable (eg, systolic blood pressure), analysis metric (eg, change from baseline, final value, time to event), method of aggregation (eg, median, proportion), and time point for each outcome. Explanation of the clinical relevance of chosen efficacy and harm outcomes is strongly recommended	_____5,6______
Participant timeline	13	Time schedule of enrolment, interventions (including any run-ins and washouts), assessments, and visits for participants. A schematic diagram is highly recommended (see Figure)	__6, Figure 1__

For peer review only - http://bmjopen.bmj.com/site/about/guidelines.xhtml

Sample size	14	Estimated number of participants needed to achieve study objectives and how it was determined, including clinical and statistical assumptions supporting any sample size calculations	_____8.9______
Recruitment	15	Strategies for achieving adequate participant enrolment to reach target sample size	_____8.9______

Methods: Assignment of interventions (for controlled trials)

Allocation:

Sequence generation	16a	Method of generating the allocation sequence (eg, computer-generated random numbers), and list of any factors for stratification. To reduce predictability of a random sequence, details of any planned restriction (eg, blocking) should be provided in a separate document that is unavailable to those who enrol participants or assign interventions	______7_______
Allocation concealment mechanism	16b	Mechanism of implementing the allocation sequence (eg, central telephone; sequentially numbered, opaque, sealed envelopes), describing any steps to conceal the sequence until interventions are assigned	_____7________
Implementation	16c	Who will generate the allocation sequence, who will enrol participants, and who will assign participants to interventions	_____7________
Blinding (masking)	17a	Who will be blinded after assignment to interventions (eg, trial participants, care providers, outcome assessors, data analysts), and how	_____7________
	17b	If blinded, circumstances under which unblinding is permissible, and procedure for revealing a participant's allocated intervention during the trial	_____7________

Methods: Data collection, management, and analysis

Data collection methods	18a	Plans for assessment and collection of outcome, baseline, and other trial data, including any related processes to promote data quality (eg, duplicate measurements, training of assessors) and a description of study instruments (eg, questionnaires, laboratory tests) along with their reliability and validity, if known. Reference to where data collection forms can be found, if not in the protocol	____6_________
	18b	Plans to promote participant retention and complete follow-up, including list of any outcome data to be collected for participants who discontinue or deviate from intervention protocols	_____6________

Data management	19	Plans for data entry, coding, security, and storage, including any related processes to promote data quality (eg, double data entry; range checks for data values). Reference to where details of data management procedures can be found, if not in the protocol	___NA_________
Statistical methods	20a	Statistical methods for analysing primary and secondary outcomes. Reference to where other details of the statistical analysis plan can be found, if not in the protocol	____7,8________
	20b	Methods for any additional analyses (eg, subgroup and adjusted analyses)	____7,8________
	20c	Definition of analysis population relating to protocol non-adherence (eg, as randomised analysis), and any statistical methods to handle missing data (eg, multiple imputation)	____7,8_______
**Methods: Monitoring**			
Data monitoring	21a	Composition of data monitoring committee (DMC); summary of its role and reporting structure; statement of whether it is independent from the sponsor and competing interests; and reference to where further details about its charter can be found, if not in the protocol. Alternatively, an explanation of why a DMC is not needed	_____NA______
	21b	Description of any interim analyses and stopping guidelines, including who will have access to these interim results and make the final decision to terminate the trial	_____8.9______
Harms	22	Plans for collecting, assessing, reporting, and managing solicited and spontaneously reported adverse events and other unintended effects of trial interventions or trial conduct	_____8,9_______
Auditing	23	Frequency and procedures for auditing trial conduct, if any, and whether the process will be independent from investigators and the sponsor	_____NA_______
**Ethics and dissemination**			
Research ethics approval	24	Plans for seeking research ethics committee/institutional review board (REC/IRB) approval	_______10_____
Protocol amendments	25	Plans for communicating important protocol modifications (eg, changes to eligibility criteria, outcomes, analyses) to relevant parties (eg, investigators, REC/IRBs, trial participants, trial registries, journals, regulators)	_______NA_____

Consent or assent	26a	Who will obtain informed consent or assent from potential trial participants or authorised surrogates, and how (see Item 32)	_____4_______
	26b	Additional consent provisions for collection and use of participant data and biological specimens in ancillary studies, if applicable	_____4______
Confidentiality	27	How personal information about potential and enrolled participants will be collected, shared, and maintained in order to protect confidentiality before, during, and after the trial	_____NA______
Declaration of interests	28	Financial and other competing interests for principal investigators for the overall trial and each study site	_____14_____
Access to data	29	Statement of who will have access to the final trial dataset, and disclosure of contractual agreements that limit such access for investigators	_____14______
Ancillary and post-trial care	30	Provisions, if any, for ancillary and post-trial care, and for compensation to those who suffer harm from trial participation	_____NA______
Dissemination policy	31a	Plans for investigators and sponsor to communicate trial results to participants, healthcare professionals, the public, and other relevant groups (eg, via publication, reporting in results databases, or other data sharing arrangements), including any publication restrictions	_____10______
	31b	Authorship eligibility guidelines and any intended use of professional writers	_____14______
	31c	Plans, if any, for granting public access to the full protocol, participant-level dataset, and statistical code	_____14_____
**Appendices**			
Informed consent materials	32	Model consent form and other related documentation given to participants and authorised surrogates	_____NA___
Biological specimens	33	Plans for collection, laboratory evaluation, and storage of biological specimens for genetic or molecular analysis in the current trial and for future use in ancillary studies, if applicable	_____NA______

*It is strongly recommended that this checklist be read in conjunction with the SPIRIT 2013 Explanation & Elaboration for important clarification on the items. Amendments to the protocol should be tracked and dated. The SPIRIT checklist is copyrighted by the SPIRIT Group under the Creative Commons "Attribution-NonCommercial-NoDerivs 3.0 Unported" license.
